# Supplementary material for: Identifying low-quality features and suboptimal models to enhance phenotype prediction
Source: Patterns (N Y). 2026 Sep 3;7(10):101643. doi: 10.1016/j.patter.2026.101643 (PMC13674310; doi:10.1016/j.patter.2026.101643)
Supplement: Supplementary file 1 [file mmc1.pdf]

Patterns, Volume *7*

Supplemental information

Identifying low-quality features and suboptimal models to enhance phenotype prediction

Baining Shen, Min Li, Xiaoyue Hu, Ming Chen, Wei-Hua Chen, and Hangjin Jiang

Supplemental Figures and Table

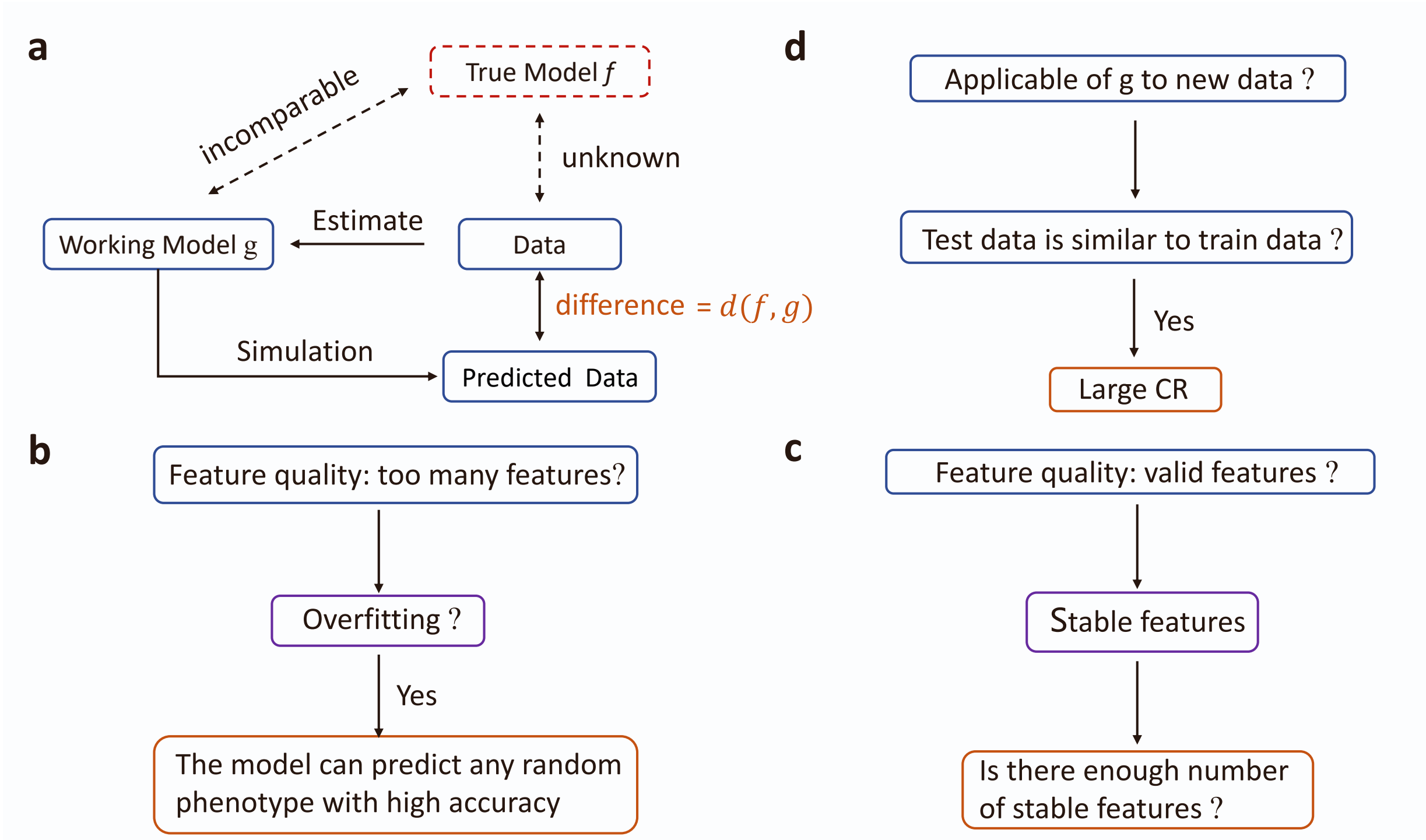

Figure S1. Conceptual summary on ideas of evaluations in mBoost. **a**, Concept of E_1 for evaluating the model structure. $d(f, g)$ is the distance between the true model f and the working model g. **b,c**, Concept of E_2(**b**), E_3(**c**) for evaluating the quality of features. **d**, Concept of E_4 on evaluating the applicability of the trained model to new dataset. Related to Fig. 1.

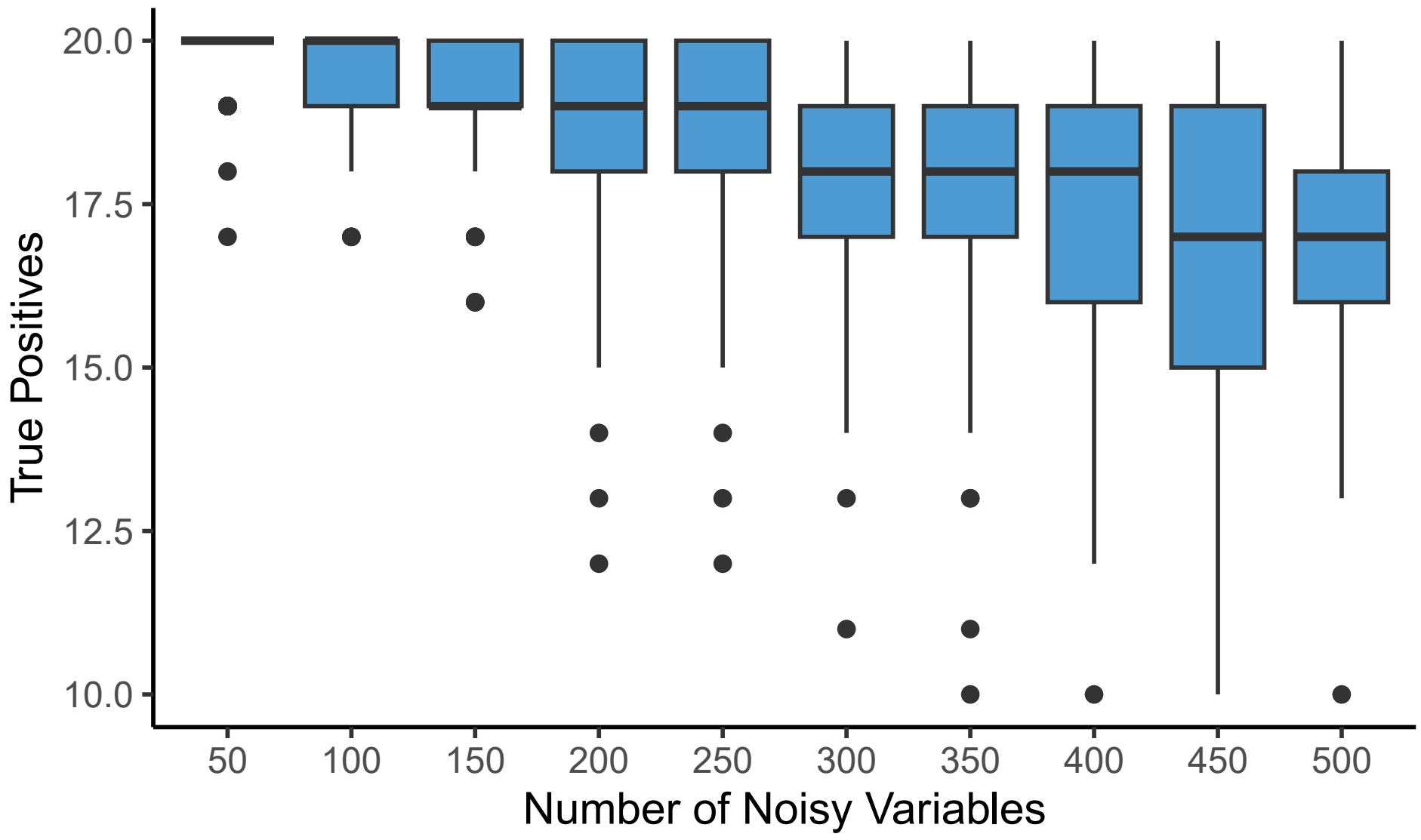

Figure S2. The feature selection capability of Lasso decreases as the number of noisy variables increases (**Methods**: Example 1). Related to Fig. 1.

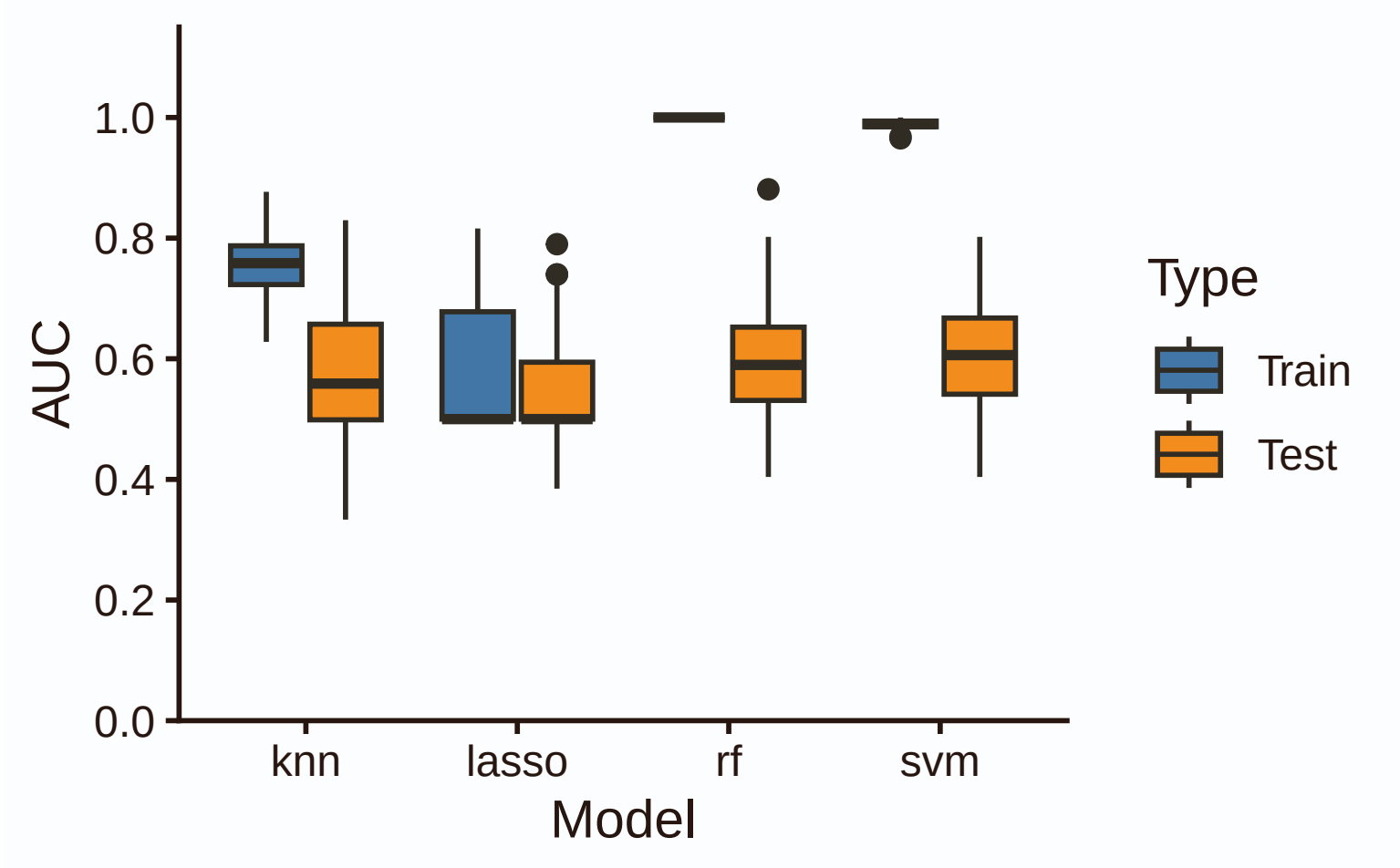

Figure S3. Modeling performance of four machine learning methods on null data. KNN and Lasso yield relatively low training AUCs, whereas Random Forest and SVM achieve near-perfect training AUCs; however, all methods produce test AUCs close to 0.5. Related to Fig. 2.

Model		KNN	Lasso	RF	SVM
Structure Improvability	I	0	0	0	0
	NI	1	1	1	1
Feature redundancy	R	0.94	0.95	1	0.95
	NR	0.06	0.05	0	0.05
Feature selection method		Wilcoxon	KS	AD	RF
Feature validity	VF	0	0	0	0
	IVF	1	1	1	1

Table S1. mBoost identifies suboptimal models and low-quality features on null data. The frequencies of the evaluation results are reported from 100 repeated simulations. mBoost consistently identified the non-improvable model structure and the low-quality features. Related to Fig. 2.

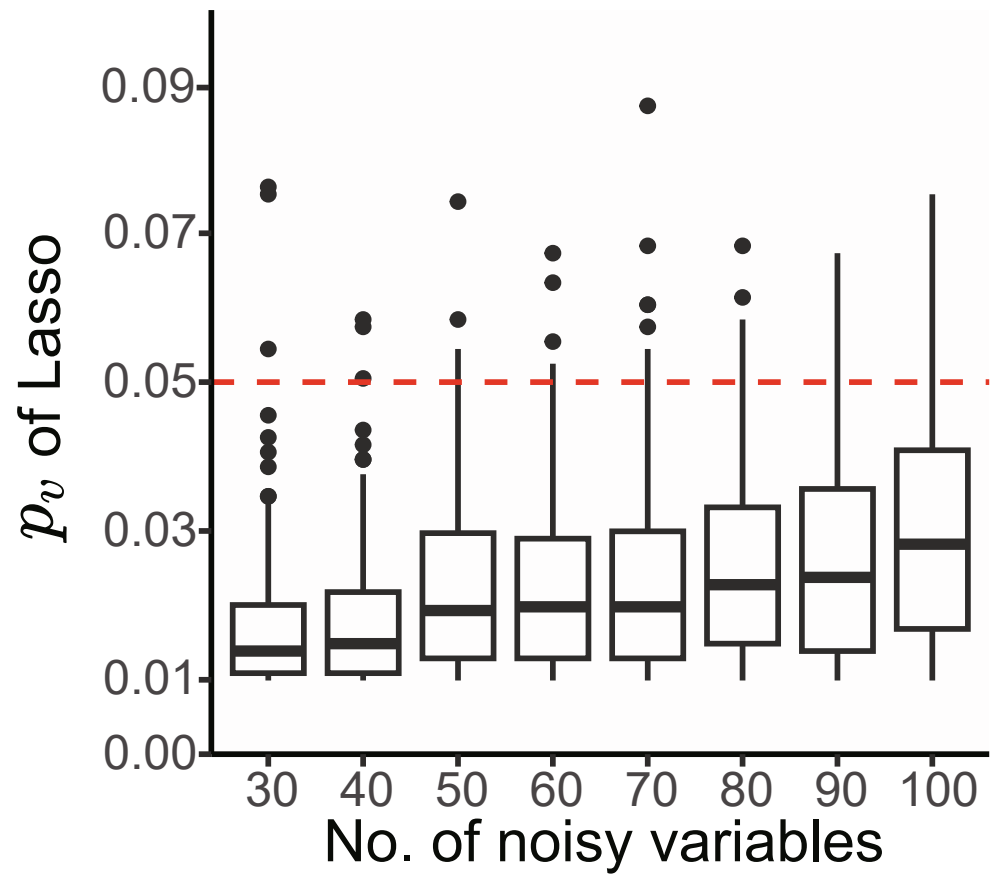

Figure S4. Lasso selects valid features in the linear logistic regression example (**Methods**: Example 4). Evaluation by mBoost revealed that in most experiments, p_v values were less than 0.05, implying that Lasso successfully identified valid features in this example. Related to Fig. 2.

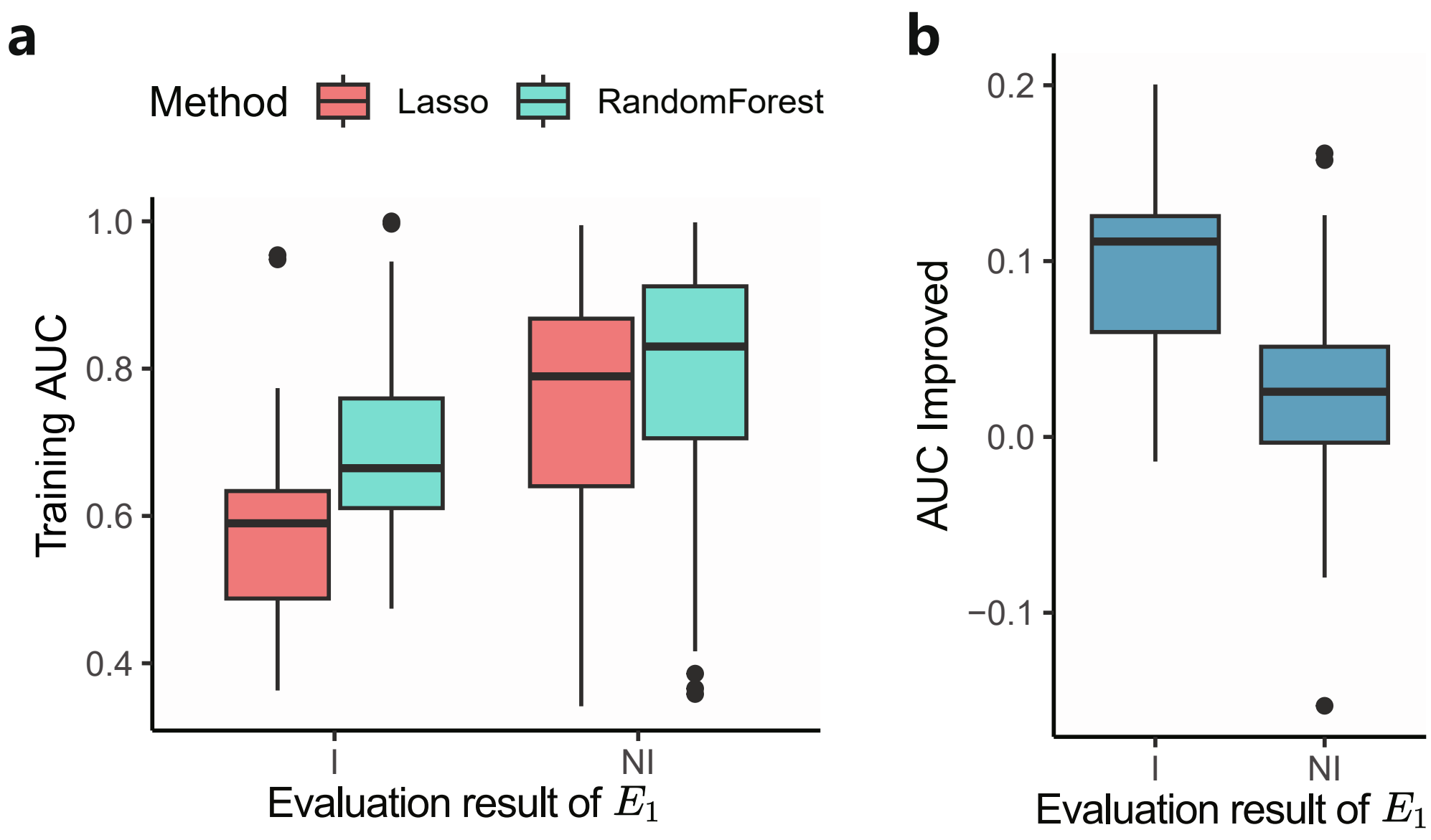

Figure S5. Internal cross-validation AUCs on the training set obtained from Lasso and RF for cohorts classified as improvable (I) and non-improvable (NI). **a**, The comparison between the training AUCs from Lasso and RF. **b**, The difference in AUCs after switching from Lasso to RF. Related to Fig. 4.

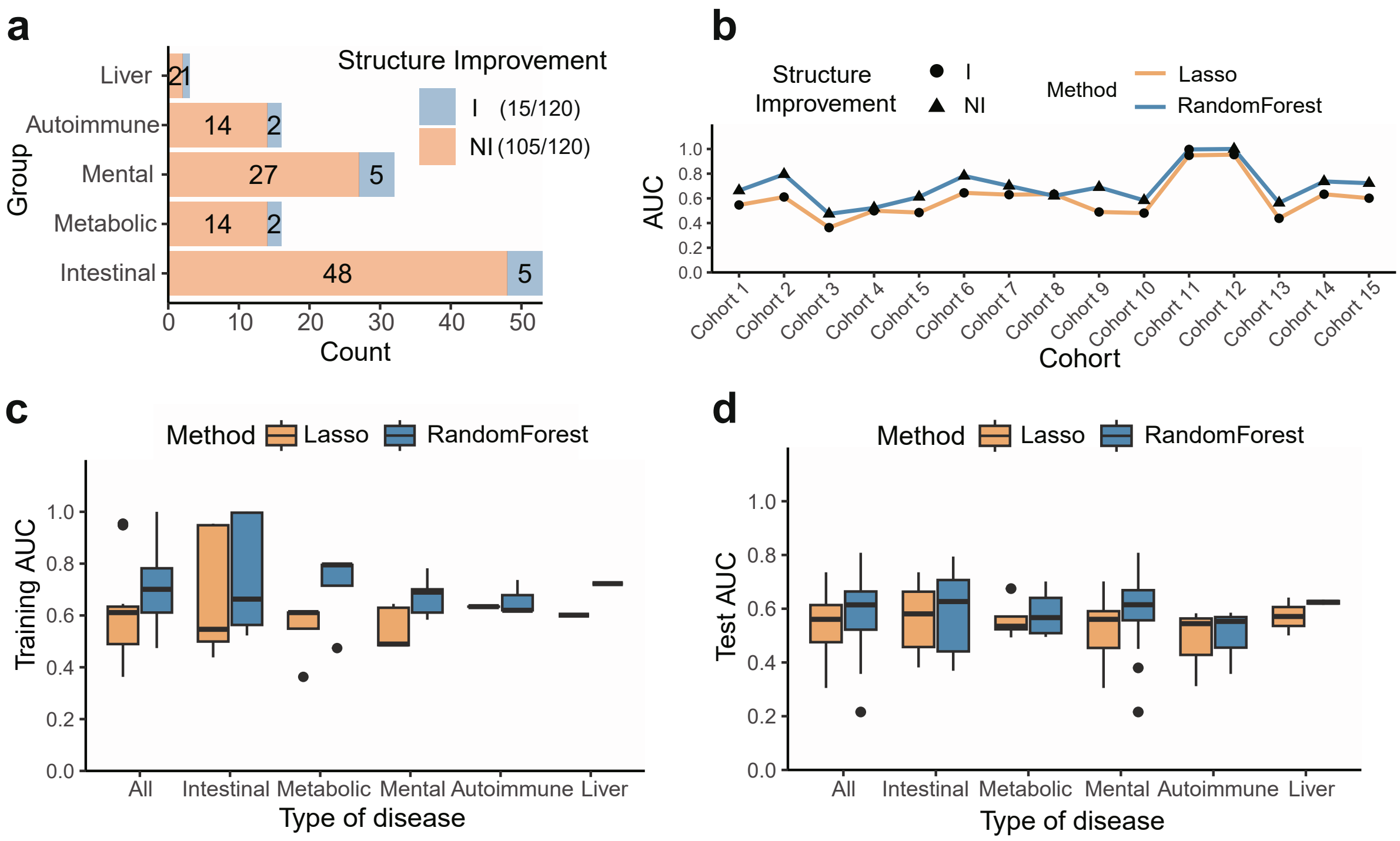

Figure S6. mBoost identifies suboptimal predictive models for different cohorts. **a**, The number of cohorts classified as **I** (improvable) and NI (non-improvable) under different disease categories when modeling microbiome data using Lasso. **b**, The AUC before and after switching to RF for improvable cohorts, and the corresponding determination results for E_1. **c**, Training AUCs before and after switching from Lasso to RF for the models identified as improvable in different disease category cohorts. **d**, Test AUC before and after switching from Lasso to RF for the models identified as improvable in different disease category cohorts. Related to Fig. 4.

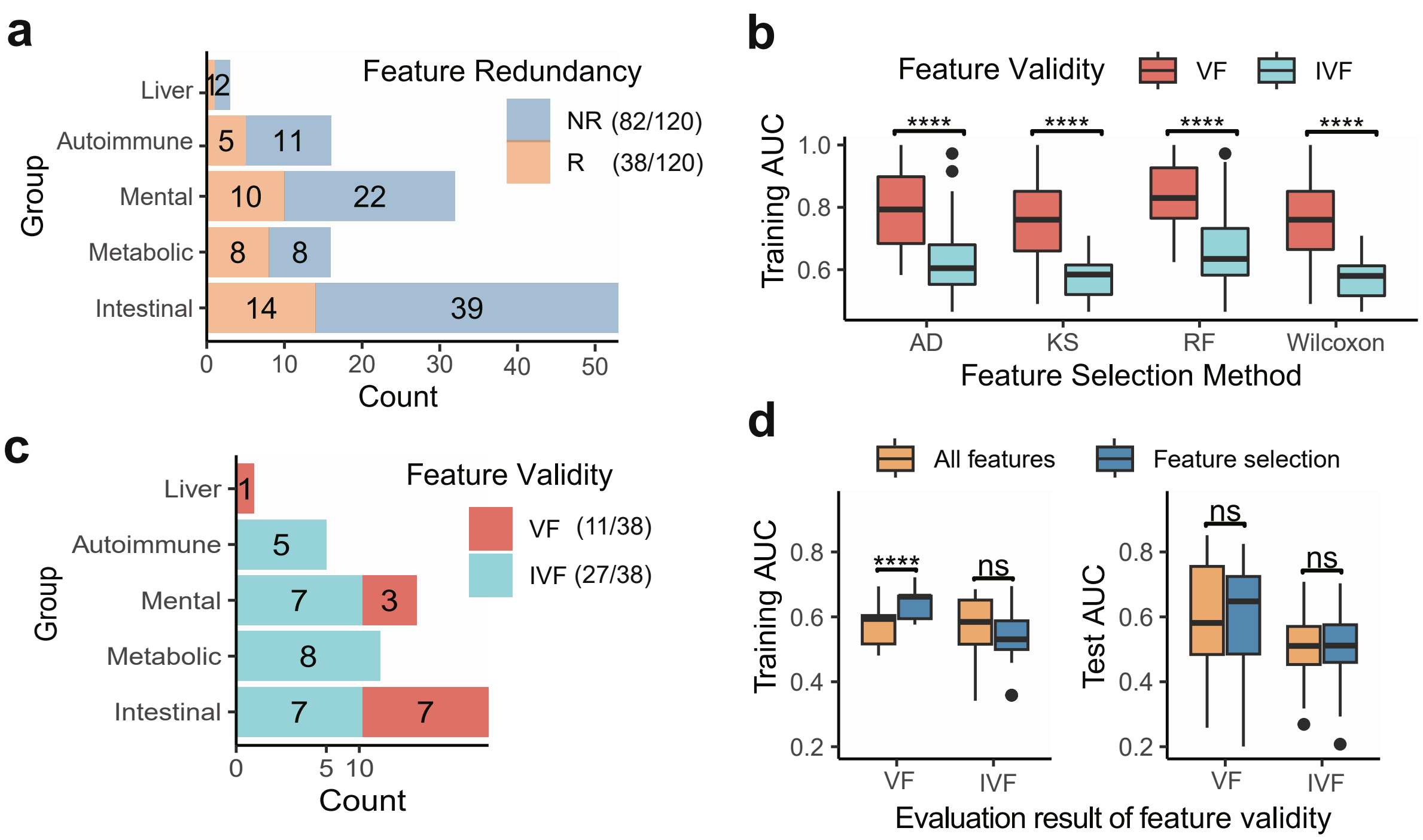

Figure S7. mBoost captures the quality of features in gut microbiome data. **a**, The number of cohorts classified as R and NR under different disease categories. **b**, Training AUCs among cohorts classified as VF and IVF. **c**, The number of cohorts classified as having valid features and those without valid features in different disease categories when using the Wilcoxon test for feature selection. **d**, Internal training AUC and external test AUC for cohorts with and without valid features. "All features" corresponds to using all of the features to build the model, and "Feature selection" corresponds to using Wilcoxon test for feature selection. For panels **b** and **d**, p values were calculated using t-tests; **** indicates $p \leq 0.0001$ and ns indicates $p > 0.05$ (sample sizes: **b**, $n = 120$; **d**, $n = 108$). Related to Fig. 4.

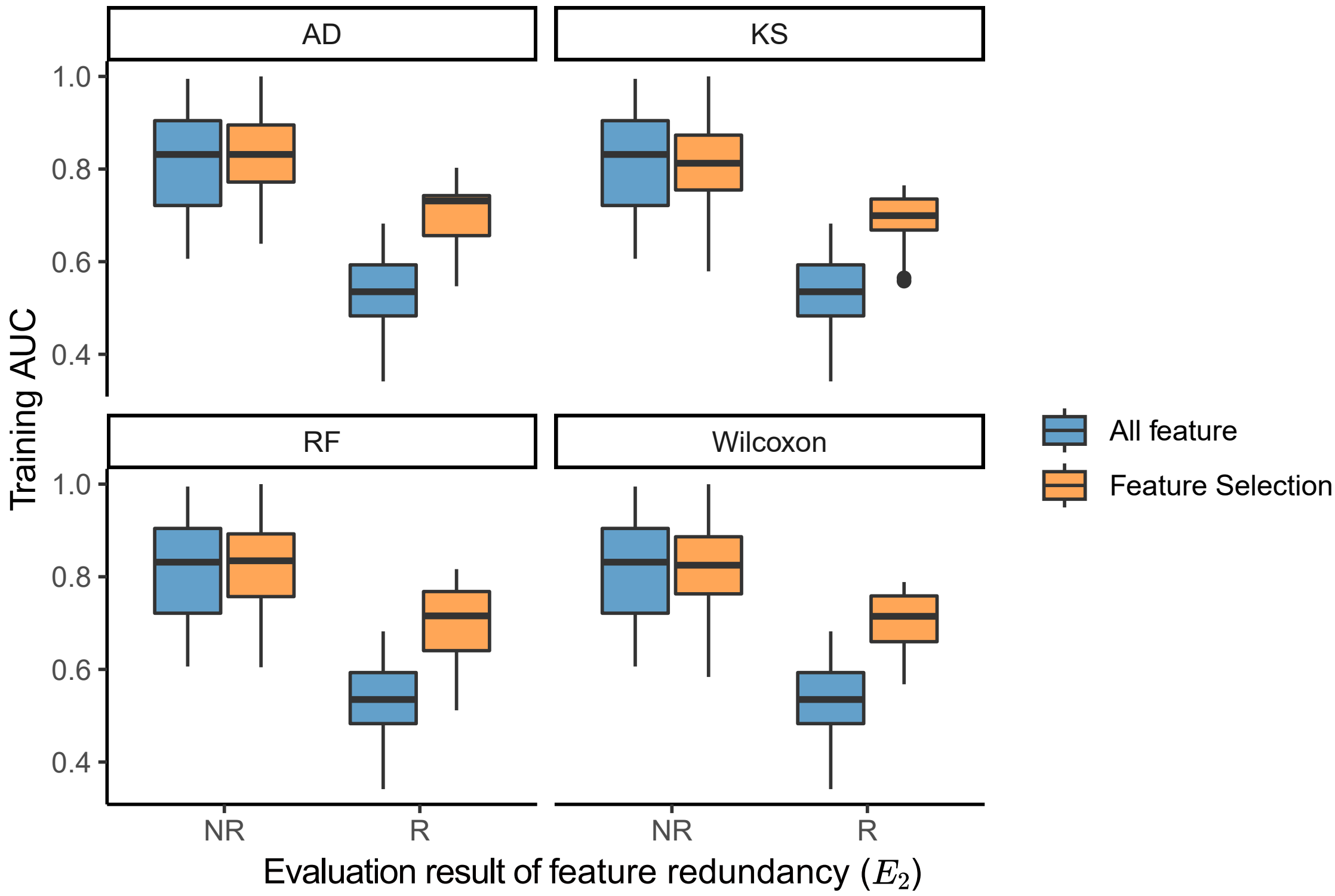

Figure S8. Training AUC before and after feature selection for cohorts classified as R and NR, where R indicates the cohorts identified as having redundant features, and NR indicates without redundant features. Four feature selection methods are used in the analysis (**Methods**). The AUC improves significantly after feature selection for R cohorts, whereas for NR cohorts the AUC shows little change before and after feature selection. "All features" means the model is trained on all features and "Feature selection" means the model is trained on selected features by different methods. Related to Fig. 4.

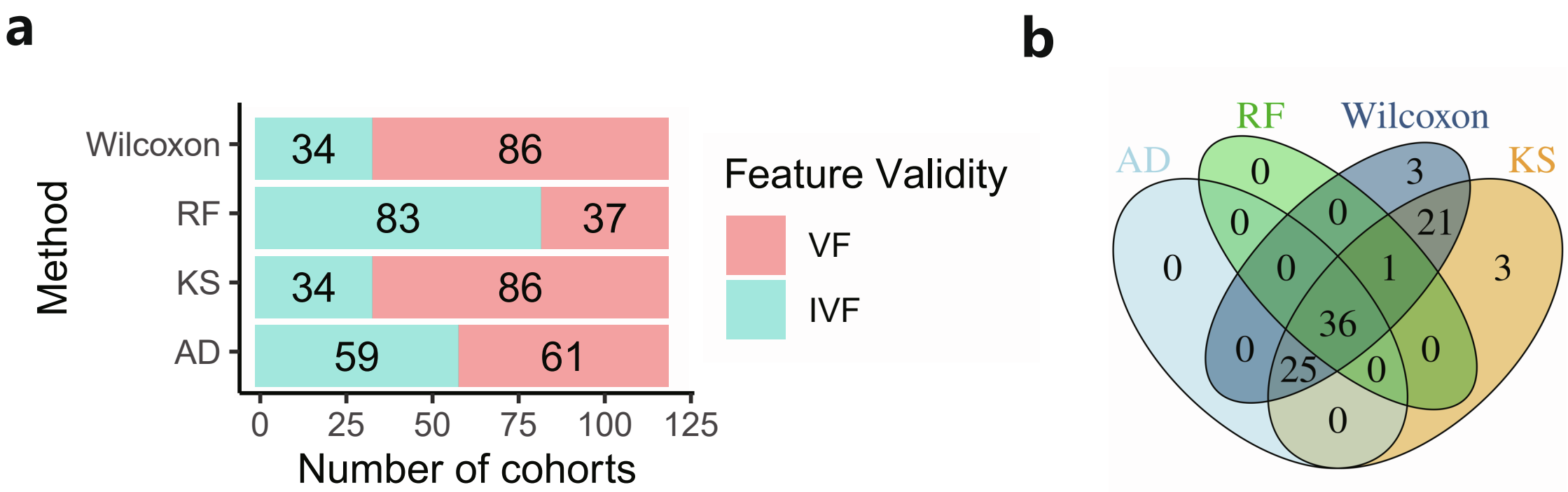

Figure S9. The distribution of cohorts identified as having valid features under four feature selection methods. **a**, The number of cohorts classified as having valid features (VF) and those without valid features (IVF). **b**, A Venn diagram illustrating the cohorts identified as having consistent features across the four feature selection methods. Related to Fig. 4.

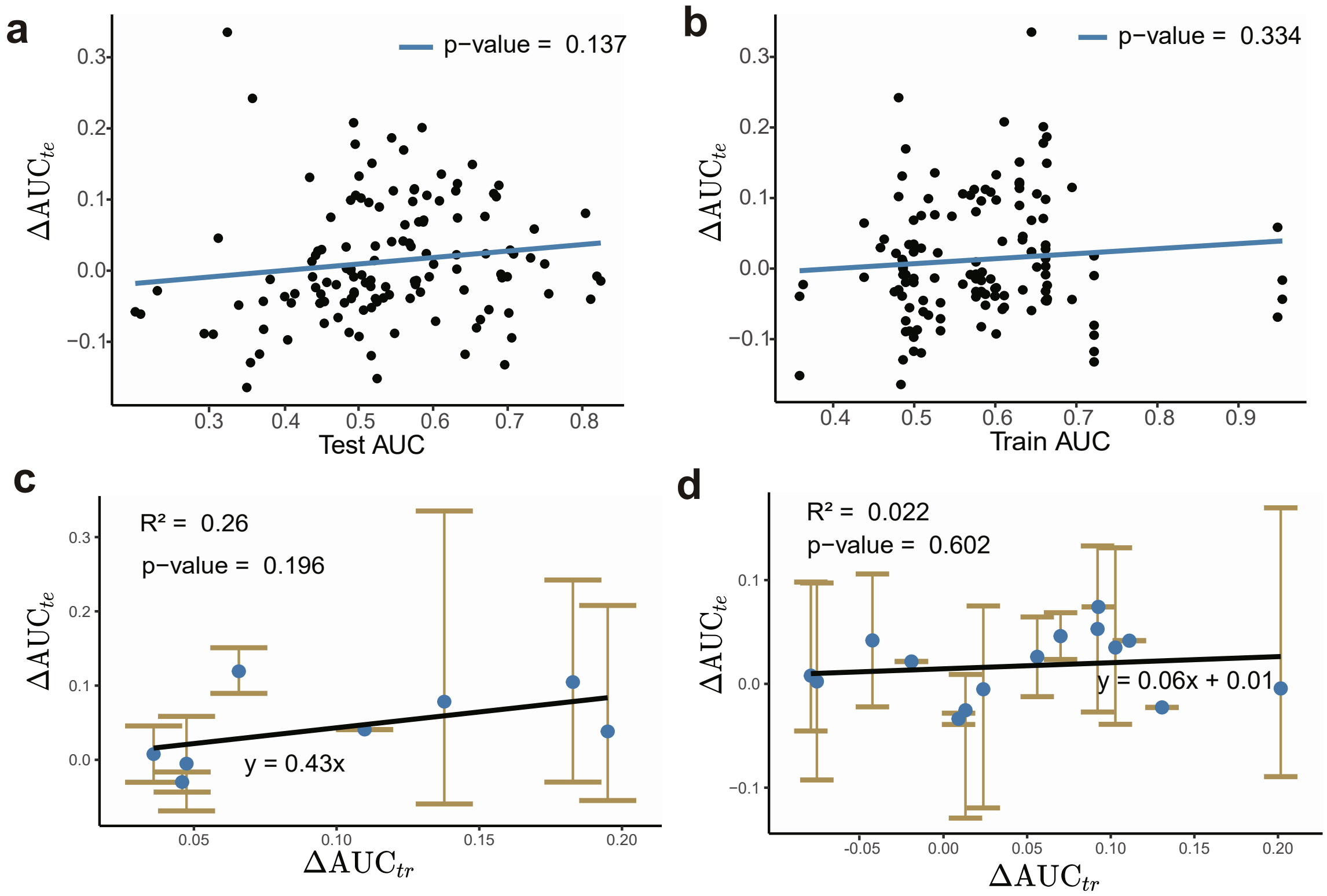

Figure S10. The relationship between ΔAUC_{te}, ΔAUC_{tr}, test AUC and training AUC, where ΔAUC_{te} is the improvement in test AUC, and ΔAUC_{tr} is the improvement in train AUC. **a**,**b**, The relationship between ΔAUC_{te} and test/training AUC on all cohorts identified as improvable. **c**,**d**, The relationship between ΔAUC_{te}, test AUC and training AUC on cohorts identified as I-NR/I-R-VF (**c**) and NI-R-IVF/I-R-IVF/NI-R-VF (**d**). P values shown in panels **a**-**d** were calculated as in Figure 4G using linear regression models fitted with R `lm()`; they correspond to two-sided t-tests for the slope (sample sizes: **a**, $n = 129$; **b**, $n = 129$; **c**, $n = 8$; **d**, $n = 15$). Related to Fig. 4.

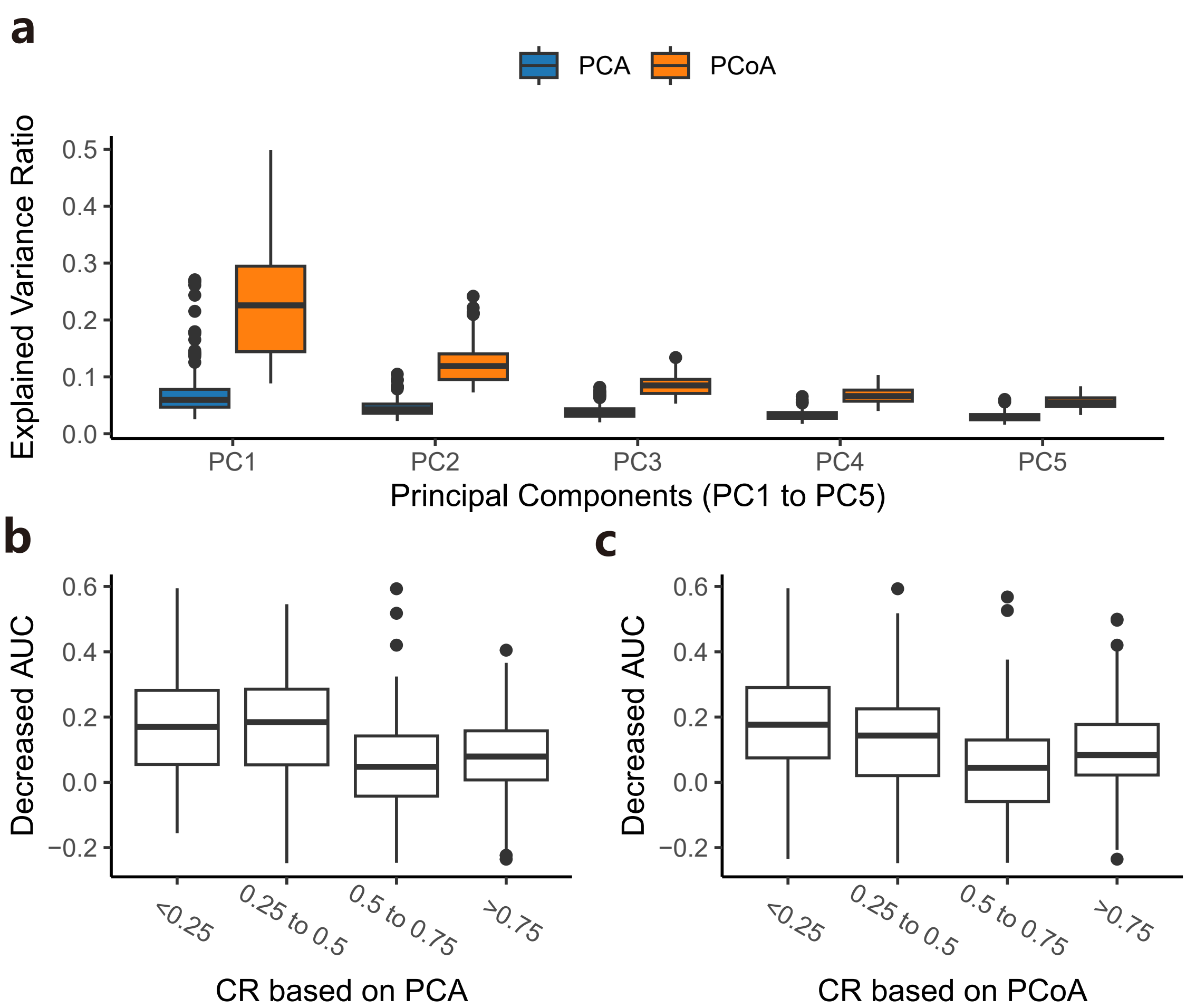

Figure S11. Coverage Ratio (CR) based on PCA and PCoA. **a**, Boxplots of the explained variance ratio for the first five principal components (PCs) from these two methods across all cohorts. **b**,**c**, The relationship between the CR based on PCA (PCoA) and the reduction in AUC. Related to Fig. 5.

Supplemental Proof

Lemma S1. *Suppose that* $\boldsymbol{U}_n = (U_{n,1}, \cdots, U_{n,k})$ *and* $\boldsymbol{V}_n = (V_{n,1}, \cdots, V_{n,k})$ *are independently sampled from multinomial distributions with parameters* $(n, a_1, \cdots, a_k)$ *and* $(n, b_1, \cdots, b_k)$*, respectively. Define the column vectors* $\boldsymbol{a} = (a_1, \cdots, a_k)$ *and* $\boldsymbol{b} = (b_1, \cdots, b_k)$*. Under the null hypothesis* H_0*:* $\boldsymbol{a} = \boldsymbol{b}$*, we have*

$$T = \sum_{i=1}^{k}\sum_{i=1}^{k} \frac{(U_{n,i} - V_{n,i})^2}{N\hat{c}_i} \to \chi^2_{k-1},$$

where $\hat{\boldsymbol{c}} = \frac{\boldsymbol{U}_n + \boldsymbol{V}_n}{2n}$ *is the estimate of* $\boldsymbol{a}$ *under* H_0*, and* $k \geq 2$*.*

Proof: Let $\{X_t\}_{t=1}^n$ and $\{Y_t\}_{t=1}^n$ be the observed category series of the count vectors $\mathbf{U}_n$ and $\mathbf{V}_n$, respectively. So we have $P(X_t = i) = a_i$ and $P(Y_t = i) = b_i$. Let $N = 2n$. We define

$$X_i^* = (X_{1,i}^*, \cdots, X_{N,i}^*) = (\delta_{i,X_1}, \cdots, \delta_{i,X_n}, 0, \cdots, 0),$$
$$Y_i^* = (Y_{1,i}^*, \cdots, Y_{N,i}^*) = (0, \cdots, 0, \delta_{i,Y_1}, \cdots, \delta_{i,Y_n}),$$

where $\delta_{i,j} = I(i = j)$.

Let $Z_i = U_{n,i} - V_{n,i} = \sum_{k=1}^N X_{k,i}^* - \sum_{k=1}^N Y_{k,i}^*$, under H_0, we have $E(Z_i) = 0, \text{Var}(Z_i) = 2na_i(1 - a_i), \text{Cov}(Z_i, Z_j) = -2na_ia_j$. So, by the multivariate central limit theorem, we have

$$\frac{1}{\sqrt{2n}}\mathbf{Z} = \frac{\mathbf{U}_n - \mathbf{V}_n}{\sqrt{2n}} \xrightarrow{d} N(0, \Sigma),$$

where $\sigma_{i,j}$, the (i,j)-th element of Σ, is equal to $a_i(\delta_{ij} - a_j)$. Since $\hat{\mathbf{c}} \xrightarrow{p} \mathbf{a}$, we have

$$\frac{\mathbf{U}_n - \mathbf{V}_n}{\sqrt{2n\hat{\mathbf{c}}}} \xrightarrow{d} N(0, I_k - \sqrt{\mathbf{a}}\sqrt{\mathbf{a}}').$$

Thus, we have $T \to \chi^2_{k-1}$, as $n \to +\infty$.

Proof of Theorem 1

Denote $\hat{p}_{ij} = P_{ij}/n$, $\hat{q}_{ij} = Q_{ij}/n$, and $\hat{r}_{ij} = \hat{p}_{ij} + \hat{q}_{ij}$ as the estimates of p_{ij}, q_{ij} and r_{ij}, respectively. Let $\Delta_{ij} = p_{ij} - q_{ij}$, and let $\hat{\Delta}_{ij} = \hat{p}_{ij} - \hat{q}_{ij}$ be its estimate.

It is well known that for $\hat{p}_{ij}$, $\hat{q}_{ij}$, we have

$$P(|\hat{p}_{ij} - p_{ij}| > \epsilon) \leq 2e^{-n\epsilon^2/2}, \quad P(|\hat{q}_{ij} - q_{ij}| > \epsilon) \leq 2e^{-n\epsilon^2/2}.$$

Thus, we have,

$$P(|\hat{\Delta}_{ij} - \Delta_{ij}| > \epsilon) \leq 4e^{-n\epsilon^2/8}, \quad P(|\hat{r}_{ij} - r_{ij}| > \epsilon) \leq 4e^{-n\epsilon^2/8}.$$

For convenience, let S be the local statistic defined in equation (3), and let S^p be its population version defined in equation (4).

$$\begin{aligned}
|S - S^p| &= n|\sum_{i=1}^{2}\sum_{j=1}^{2} \frac{(\hat{p}_{ij} - \hat{q}_{ij})^2}{\hat{r}_{ij}} - \sum_{i=1}^{2}\sum_{j=1}^{2} \frac{(p_{ij} - q_{ij})^2}{r_{ij}}| \\
&\leq n\sum_{i=1}^{2}\sum_{j=1}^{2} |\frac{(\hat{p}_{ij} - \hat{q}_{ij})^2}{\hat{r}_{ij}} - \frac{(p_{ij} - q_{ij})^2}{r_{ij}}| \\
&= n\sum_{i=1}^{2}\sum_{j=1}^{2} |\frac{(\hat{p}_{ij} - \hat{q}_{ij})^2}{\hat{r}_{ij}} - \frac{(\hat{p}_{ij} - \hat{q}_{ij})^2}{r_{ij}} + \frac{(\hat{p}_{ij} - \hat{q}_{ij})^2}{r_{ij}} - \frac{(p_{ij} - q_{ij})^2}{r_{ij}}| \\
&\leq n\sum_{i=1}^{2}\sum_{j=1}^{2} \frac{\hat{\Delta}_{ij}^2}{\hat{r}_{ij}r_{ij}}|\hat{r}_{ij} - r_{ij}| + \frac{|\hat{\Delta}_{ij}^2 - \Delta_{ij}^2|}{r_{ij}} \\
&\leq n\sum_{i=1}^{2}\sum_{j=1}^{2} \frac{|1 - r_{ij}/\hat{r}_{ij}|}{\eta} + \frac{2|\hat{\Delta}_{ij} - \Delta_{ij}|}{\eta}
\end{aligned}$$

Define the events $E_1 = \{|\hat{\Delta}_{ij} - \Delta_{ij}| > \epsilon\}$ and $E_2 = \{|\hat{r}_{ij} - r_{ij}| > \epsilon\}$. Now, on $E_1^c \bigcap E_2^c$, we have

$$|S - S^p| \leq \frac{4n\epsilon}{\eta}(\frac{1}{\eta} + 2). \tag{S1}$$

Finally, let $\delta = \frac{4n\epsilon}{\eta}(\frac{1}{\eta} + 2)$, we have

$$P(|S - S^p| > \delta) \leq 1 - P(E_1^c \bigcap E_2^c) \leq P(E_1) + P(E_2) = 8e^{-n\epsilon^2/8}. \tag{S2}$$

Thus, $P(|\text{MAC}(D, D^*) - \text{MAC}^p(D, D^*)| > \delta) \leq 16n^2e^{-n\epsilon^2/8}$, and the other two conclusions follow directly.
