# Supplementary material for: Identifying low-quality features and suboptimal models to enhance phenotype prediction
Source: Patterns (N Y). 2026 Sep 3;7(10):101643. doi: 10.1016/j.patter.2026.101643 (PMC13674310; doi:10.1016/j.patter.2026.101643)
Supplement: Supplementary file 2 [file mmc2.pdf]

Patterns

Article

Identifying low-quality features and suboptimal models to enhance phenotype prediction

Graphical abstract

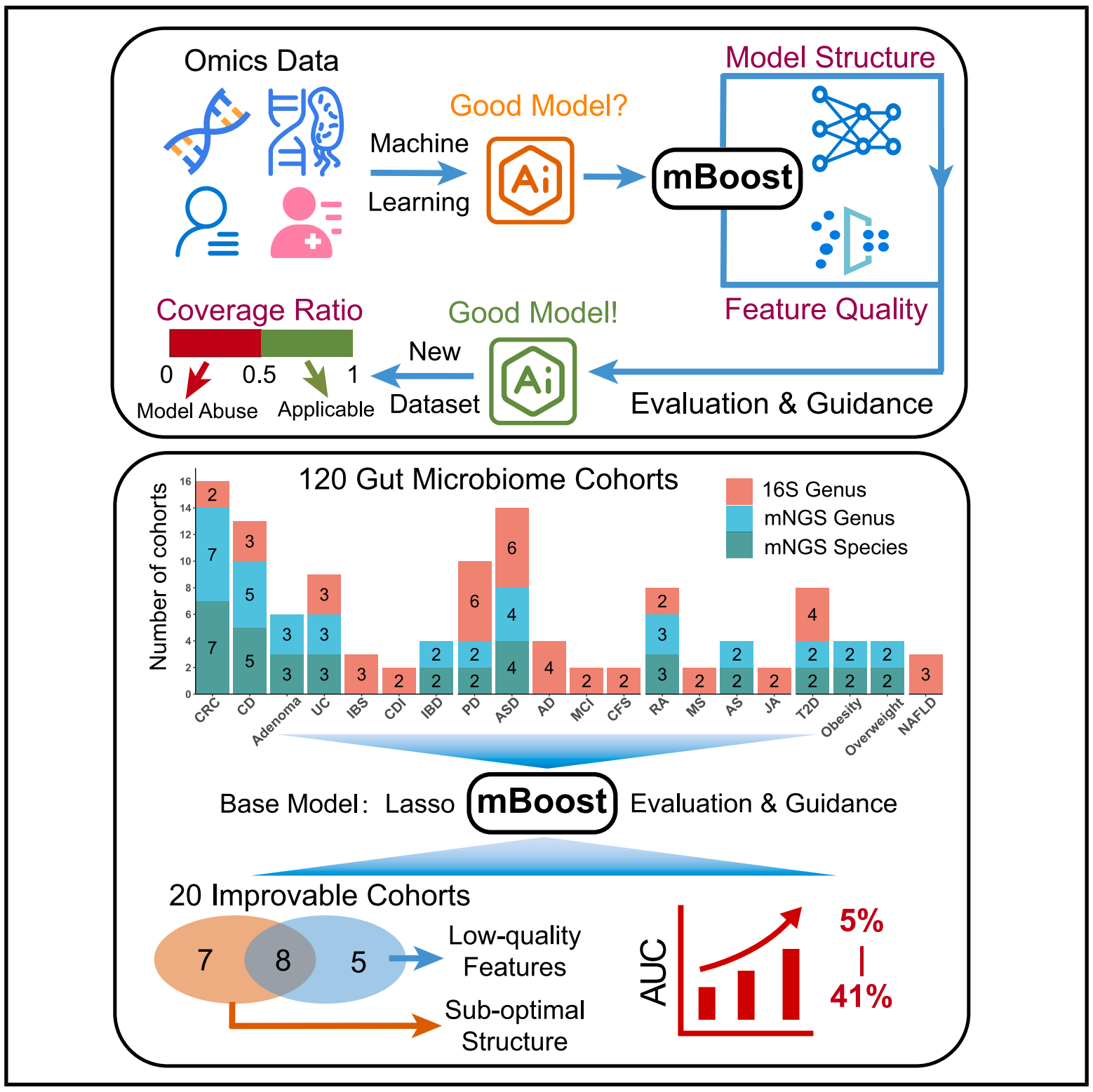

Authors

Baining Shen, Min Li, Xiaoyue Hu,
Ming Chen, Wei-Hua Chen,
Hangjin Jiang

Correspondence

weihuachen@hust.edu.cn (W.-H.C.),
jianghj@zju.edu.cn (H.J.)

In brief

Shen et al. present mBoost, a diagnostic framework for building phenotype predictive models. mBoost evaluates model structure, feature redundancy, feature validity, and applicability to new data, helping researchers decide whether to use more complex models, do feature selection, or train a new model on new dataset.

Highlights

- mBoost evaluates ML models beyond traditional accuracy metrics
- It assesses model structure, feature quality, and validity
- mBoost evaluates whether trained models are applicable to new data
- It provides step-by-step guidance to enhance model performance

Shen et al., 2026, Patterns *7*, 101643
October 9, 2026 © 2026 The Author(s). Published by Elsevier Inc.
https://doi.org/10.1016/j.patter.2026.101643

CellPress

Patterns

CellPress
OPEN ACCESS

Article

Identifying low-quality features and suboptimal models to enhance phenotype prediction

Baining Shen,[1,2,6] Min Li,[3,6] Xiaoyue Hu,[1,2] Ming Chen,[4] Wei-Hua Chen,[3,5,*] and Hangjin Jiang[2,7,*]
[1]School of Mathematical Sciences, Zhejiang University, Hangzhou 310058, China
[2]State Key Laboratory for Vegetation Structure, Function and Construction (VegLab), Center for Data Science, Zhejiang University, Hangzhou 310058, China
[3]Key Laboratory of Molecular Biophysics of the Ministry of Education, Hubei Key Laboratory of Bioinformatics and Molecular Imaging, Department of Bioinformatics and Systems Biology, Center for Artificial Intelligence Biology, Huazhong University of Science and Technology, Wuhan 430074, China
[4]College of Life Sciences, Zhejiang University, Hangzhou 310058, China
[5]School of Biological Science, Jining Medical University, Rizhao 276800, China
[6]These authors contributed equally
[7]Lead contact
*Correspondence: weihuachen@hust.edu.cn (W.-H.C.), jianghj@zju.edu.cn (H.J.)
https://doi.org/10.1016/j.patter.2026.101643

THE BIGGER PICTURE Biomedical researchers increasingly rely on machine learning to predict disease outcomes and treatment responses from omics and clinical data. Yet, these models are often developed using small or varied patient groups, and this makes it difficult to know whether a model has learned meaningful biological patterns or has simply fit the available data. Standard measures of predicting accuracy do not always reveal whether a model is too simple, unnecessarily complex, built with uninformative features, or likely to perform well with different datasets. mBoost addresses these challenges by introducing a framework for examining these issues. Based on this assessment, mBoost can help researchers decide when to adjust the model, refine features, or train a new model for a different dataset.

SUMMARY

It is common to build predictive models for complex phenotypes, such as diseases, where sample sizes are often limited by the cost of data collection. Machine learning and statistical methods are widely used in these cases. However, they may fail to identify high-quality features and lead to suboptimal models with high underfitting or overfitting risks. Here, we present mBoost to identify low-quality features and suboptimal models to enhance phenotype prediction. Importantly, it provides confidence on model performance, helping researchers avoid the frustrating cycle of model switching for possibly better performance. We demonstrate mBoost with simulations and real-data examples. Specifically, mBoost identifies optimal depth and width for a deep neural network in a drug example, detects suboptimal models in microbiome cohorts, and proposes a measure for telling whether the trained model is applicable to new data. Thus, mBoost will serve as a practical tool for model building and diagnosis.

INTRODUCTION

The prediction of complex traits is crucial in both biological and clinical research. The development of omics technologies provides opportunities to gain a deeper understanding of the molecular mechanisms underlying complex diseases. This also enables us to predict diseases based on omics data, such as the gut microbiome,[1,2] RNA sequencing (RNA-seq),[3,4] proteomics,[5–7] metabolomics,[8,9] and DNA methylation.[10] For example, gut microbiome data are widely used for disease stratification because the alteration of gut microbiome in patients, compared with healthy controls, has been increasingly used for non-invasive disease pre-screening and diagnosis[2] because of its association with many diseases,[11,12] such as colorectal cancer (CRC),[13] liver cirrhosis (LC),[14] and autism spectrum disorder (ASD).[15,16] The gut microbiome community comprises hundreds of species, but only a few of them are present in almost all samples. According to results from population cohorts,[17] the majority of taxa are present in less than 50% of the samples, which poses a great challenge in identifying biomarkers.

Despite their inherent challenges,[18] machine learning (ML) is a promising method to build diagnostic models for complex

This is an open access article under the CC BY license (http://creativecommons.org/licenses/by/4.0/).

phenotypes.[19,20] However, the performance of ML models is often limited due to some confounding factors. For example, a gut microbiome-based predictive model can be strongly affected by the intrinsic heterogeneity of gut dysbiosis across cohorts, limited clinical samples, and biases introduced by lifestyle,[21] diet,[22] drugs,[23,24] regional differences,[25] and sample processing methods.[26] Several studies have proposed methods to enhance the performance of ML models for diseases.[19] These methods mainly involve comparing the performance of different procedures and selecting the one with the best performance.[27] However, this often results in a frustrating cycle of switching to more sophisticated models without offering mechanistic insights into the key factors impacting the model performance or specific optimization strategies.

To address these challenges, we develop mBoost, a new statistical framework, to comprehensively evaluate ML predictors for complex traits and to provide step-by-step guidance for building predictive models. mBoost evaluates models from two critical aspects: whether more complex models (e.g., random forest [RF] and deep learning compared with linear models) are necessary and whether the covariates (features) are of high quality. Finally, we also consider whether the model is applicable to new datasets. Consequently, mBoost introduces comprehensive approaches for goodness-of-fit test, feature quality assessment, and evaluation of model applicability to new datasets. We validate the efficacy of mBoost through extensive simulations and application to 120 case-control gut microbiome cohorts encompassing 20 diseases, totaling 9,708 samples. Remarkably, mBoost identifies suboptimal models for different cohorts by evaluating the model performance in terms of their model structures and feature quality. Our results demonstrate that mBoost facilitates performance gains ranging from 5% to 41% for suboptimal cases. We believe that mBoost will serve as an important tool for model evaluation and performance enhancement.

RESULTS

Overview of mBoost

mBoost is a statistical framework designed to comprehensively evaluate the performance of ML models during the training and test stages, and it also provides guidance on how to improve those models when necessary. Specifically, mBoost evaluates the performance of the ML models in terms of model structure and feature quality (Figure 1). At each stage, we develop statistical tests to assess whether the model can be improved and, if so, recommend optimization strategies.

First, we evaluate whether the model structure can be improved further, asking whether a more complex structure could improve model performance. Following the idea of predictive model checking,[28,29] we assess model quality by comparing the distribution of observations from the working model with that from the true model (Figure S1A; see methods), utilizing our previously defined maximum adjusted chi-squared (MAC) statistics for two-sample distribution comparisons.[30] Figure 1B gives an illustration of the testing procedure, and a significant result, indicated by a p value $p_s \leq 0.05$, suggests that a more complex model is beneficial.

Noisy variables that are independent of the phenotype have a negative impact on the performance of the model and the feature selection methods. For example, the performance of LASSO on selecting valuable variables is negatively correlated with the number of noisy variables in the dataset (see methods: example 1; Figure S2). Thus, for feature quality testing, we first check whether the features are redundant in a model. If yes, the model tends to overfit and achieves a high training accuracy on data with a randomly permuted phenotype. On the basis of this observation, we propose our test procedure for feature redundancy (Figure 1C and methods). A p value $p_r > 0.05$ indicates redundant features. In this case, we recommend performing feature selection using methods like LASSO,[31] ElasticNet,[32] and others.[33] However, non-redundant features are not necessarily valid features that truly contribute to the phenotype. This motivates us to measure feature validity by using stable features,[34] i.e., features that are stable across different subsets of the dataset. Specifically, we randomly divide the dataset into two parts and measure the number of stable features, i.e., the size of overlap between the selected features from these two parts (Figure 1D; methods). If the decision p value is $p_v \leq 0.05$, the features are valid. Otherwise, we suggest to use other feature selection methods. If many methods give invalid features, it suggests that these features are likely poor predictors of the phenotype.

mBoost identifies suboptimal models and low-quality features in simulations

We first demonstrate the power of mBoost in identifying suboptimal models with several simulated datasets. First, we focus on a polynomial dataset whose true model is a quadratic function (methods: example 2; Figure 2A). When we fit the data with a simple linear model, mBoost yields a significant p value ($p_s = 0.01$). This rejects the null hypothesis that the linear model is good enough for the data (Figure 2A). Thus, we use a more complex model, a quadratic model, to fit the same data and obtain a non-significant p value ($p_s = 0.43$). This indicates that the model is acceptable, and there is no need to pursue more complex models, consistent with the ground truth. Second, we focus on a dataset with crescent-shaped distribution (methods: example 3; Figure 2B). In this example, we train a linear logistic model and obtain a test accuracy of 0.91 with $p_s = 0.018$. This indicates that the linear logistic model is not good enough, even though its accuracy is very high. Next, we train a kernel SVM and a RF, obtaining test accuracies of 0.98 ($p_s = 0.81$) and 0.99 ($p_s = 0.88$), respectively. This suggests these two models cannot be improved further. In summary, mBoost correctly identifies the kernel SVM as a better alternative, which underscores its sensitivity and the fact that model accuracy is independent of its optimality.

We next evaluate the power of mBoost in identifying redundant and invalid features. To this end, we simulate data from a linear logistic regression model with different numbers of noisy variables (methods: example 4). Typically, the training accuracy increases with the number of noisy variables, and feature redundancy is identified when there are over 70 noisy variables. Notably, the test accuracy declines rapidly thereafter—this substantiates the validity of our judgment that noisy variables will lead to model overfitting (Figure 2C, left). In contrast, when LASSO is used for feature selection and modeling, both the training and test sets show stable performance, and mBoost also identifies no feature redundancy (Figure 2C, right). In

CellPress
OPEN ACCESS

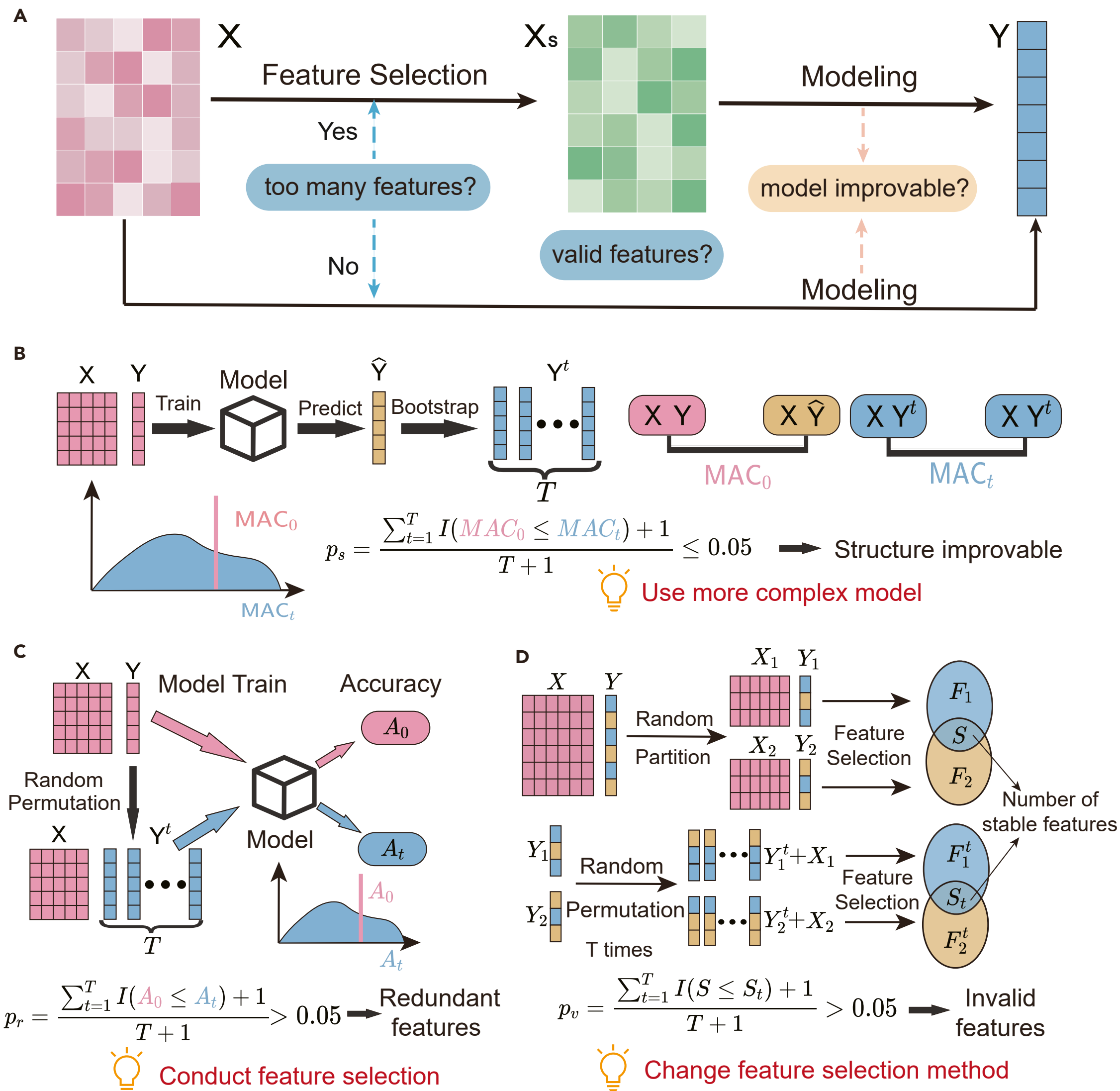

Figure 1. The framework of mBoost

(A) Two important issues in building predictive models: model quality and feature quality in terms of feature redundancy (are there too many features?) and feature validity (do these features truly contribute to the phenotype?).

(B) Testing procedure for evaluating the possibility of improving the working model with decision p value p_s, where $\hat{Y}$ is a predicted phenotype from the working model. MAC is a statistic used to measure the distance between the true model and the working model (see methods). If $p_s \leq 0.05$, it means that the structure is improvable (denoted as I) and a more complex model is preferred; otherwise, the structure is considered non-improvable (denoted as NI).

(C) An illustration of testing feature redundancy with decision p value p_r. If $p_r > 0.05$, it means there are redundant features (denoted as R), and feature selection is recommended; otherwise, features are considered non-redundant (denoted as NR).

(D) An illustration of testing the validity of features, with decision p value p_v. If $p_v \leq 0.05$, it means that the features are valid (denoted as VF); otherwise, the features are considered invalid (denoted as IVF) and we recommend using another feature selection method to select valid features.

summary, mBoost correctly evaluates feature redundancy. However, p_r does not report feature redundancy until the dataset contains 70 noisy variables (Figure 2C, left), consistent with our claim that non-redundant features are not necessarily valid features that truly contribute to the phenotype. Comparatively, LASSO always selects valid features in this example, as indicated by a small p_v (Figure S4). These results reveal different roles played by p_r and p_v.

To further clarify the distinction between feature redundancy and feature validity, we focus on a nonlinear classification example (methods: example 5), whose true pattern is shown in Figure 2D. The first two features of the simulated data have a nonlinear relationship with the classification label, while the remaining features are all noisy variables. We evaluate model performance under different settings on the number of noisy variables (ranging from 98 to 498) involved in the model. Results are reported from 100 repetitions for each setting. Figure 2E shows the training and test AUC (area under the receiver operating characteristic curve) values for the RF model and the corresponding feature evaluation results derived from p_r and p_v.

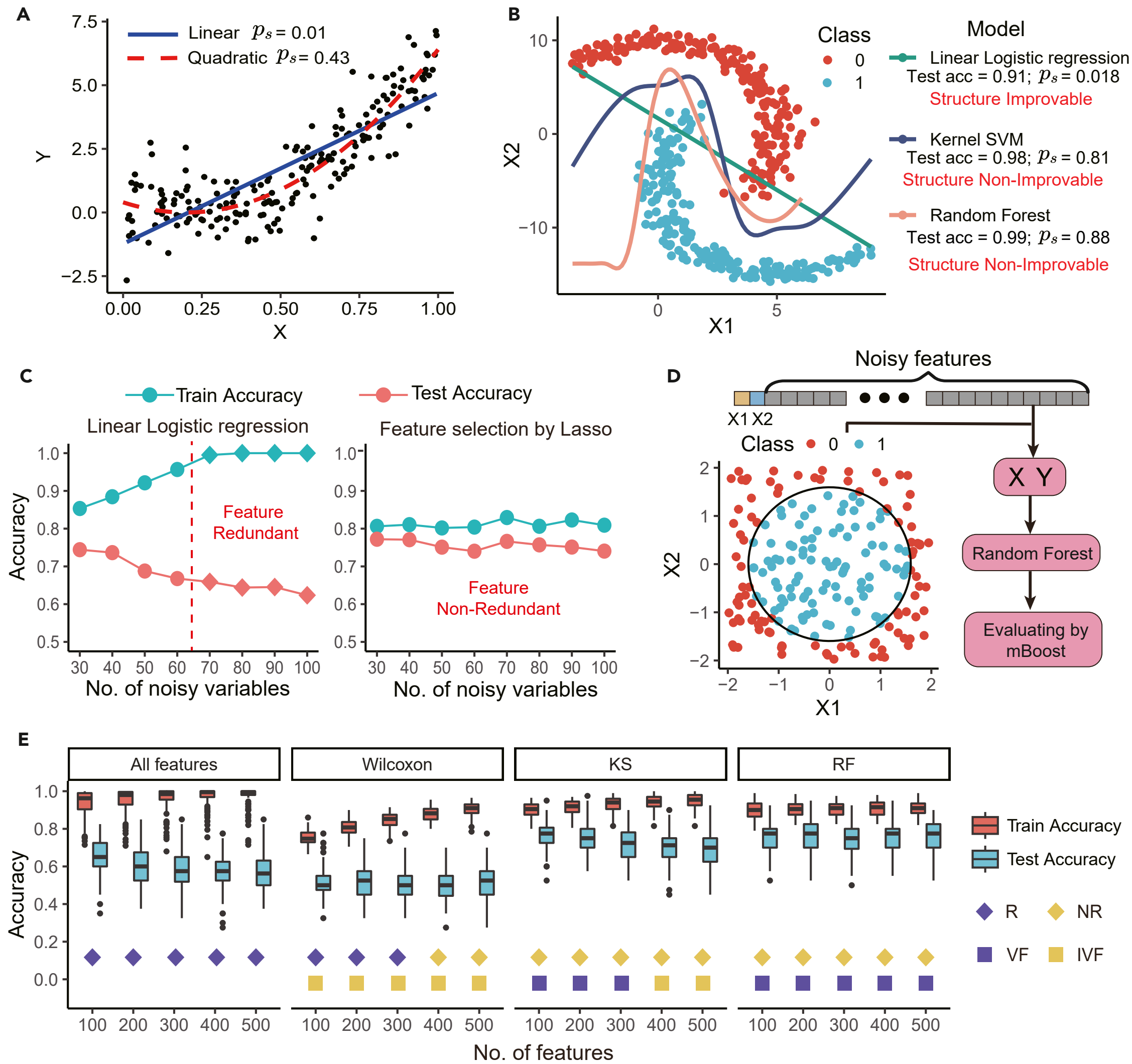

Figure 2. mBoost identifies suboptimal models and low-quality features in simulations

(A) A simulation example with a quadratic relationship between *X* and *Y* (example 2: polynomial regression). The *p* value for a linear model is $p_s = 0.01$, suggesting it is improvable; the *p* value for a quadratic model is $p_s = 0.43$, suggesting it cannot be improved further.

(B) Crescent-shaped classification example (example 3: crescent-shaped classification). First, a linear logistic model is trained, and we get a test accuracy of 0.91 with $p_s = 0.018$. This indicates that the linear logistic model is not good enough, even though its accuracy is very high. Next, a kernel SVM and RF are trained, and we get test accuracies of 0.98 ($p_s = 0.81$) and 0.99 ($p_s = 0.88$), respectively. This suggests that these two models cannot be improved further. Thus, a kernel SVM is an efficient model for this example.

(C) A linear logistic regression example (example 4: linear logistic regression) used to explore the behavior of feature redundancy along with the number of noisy variables. With the increasing number of noisy variables, the linear logistic model tends to be overfitting due to feature redundancy, which is avoided when LASSO is applied.

(D) Schematic of the workflow for the nonlinear example (example 5: feature selection in a nonlinear problem). The first two features of the simulated data have a nonlinear relationship with the classification label, while the remaining features are all noisy variables.

(E) Training and test accuracy obtained using RF from 100 repetitions, along with the corresponding evaluation results for feature redundancy and validity. When no feature selection is performed, feature redundancy is identified; in contrast, applying different feature selection methods yields distinct identification results, which are consistent with the model performance on the test set.

Initially, RF reports in generally high training AUC but low test AUC under different settings (Figure 2E, first image). mBoost reports $p_r > 0.05$, indicating redundant features. Thus, we apply three feature selection methods including Wilcoxon test, Kolmogorov-Smirnov (KS) test, and RF (see methods) to the datasets. As expected, applying feature selection procedures significantly decreases the feature redundancy. However, only the features selected by RF are valid because it always yields smaller p_v values with $p_v \leq 0.05$, which is also evidenced by a smaller difference between the training and test performance (Figure 2E). These results confirm the power of p_r and p_v in identifying the quality of features and further confirm our claim that non-redundant features may also be invalid. Additionally, they highlight that the choice of feature selection method can

significantly impact subsequent modeling outcomes, underscoring the importance of a careful evaluation of feature selection approaches. The point is that each feature selection method will reduce feature redundancy, but only some of them may provide valid features.

Finally, we conduct negative control experiments on the simulated null data to confirm the reliability of mBoost (methods: example 6). When applied to datasets without true signals, mBoost accurately and consistently diagnoses that the trained models (KNN, Lasso, RF, and SVM) are acceptable. However, a big difference between training and test accuracy tells the risk of overfitting of these models (Figure S3). Evaluating on feature quality, mBoost correctly identifies the presence of information redundancy, and all feature selection methods (Wilcoxon, KS, Anderson-Darling [AD], and RF) give invalid features (Table S1). Thus, we conclude that we cannot build a model on the null data, consistent with the ground truth.

mBoost provides valuable recommendations on building a deep neural network for drug sensitivity prediction

Previously, Chawla et al.[35] established a framework for predicting cancer drug sensitivity by integrating multi-omics data with chemical information. Their approach began with RNA-seq gene expression profiles for 550 cell lines from the Cancer Cell Line Encyclopedia (CCLE). Subsequently, these profiles were transformed using gene set variation analysis (GSVA) to generate 1,329 pathway activity scores, effectively representing the biological state of each cell line. For drug features, 173 compounds were encoded using their SMILES representations to produce SMILESVec descriptors, each as a vector of size 100. The integration of these sources resulted in a final CCLE training dataset constituting 80,056 cell line-drug combinations in rows, with 1,429 features (1,329 pathways and a 100-dimensional drug vector) as the explanatory variables and the natural logarithm of the IC_{50} value (denoted as IC_{50}) as the response variable. This dataset was then partitioned into a 90% training set (72,262 cell line-drug pairs) and a 10% test set (7,794 cell line-drug pairs), ensuring no overlap in the cell lines between these two sets. To model the complex relationships within these data, we developed Precily, a deep neural network (DNN), for the regression task of predicting drug response.

While Precily demonstrates high predictive accuracy, the performance of such deep learning models depends on the selection of hyper-parameters including the width and depth of the neural network. This selection process often relies on computationally intensive and time-consuming methods, such as extensive grid search, which can be a significant bottleneck in model development. mBoost offers a more effective way to select these hyper-parameters for DNNs.

Following the Precily framework, the input layer of the initial DNN is set to 1,429 neurons, followed by a fixed hidden layer of 512 neurons. All subsequent hidden layers use the ReLU activation function and are regularized with a dropout rate of 0.2 (Figure 3A). To isolate the effect of network architecture, we fix the learning rate at 0.001 and confine the hyper-parameter search to network depth and width. Specifically, we evaluate architectures varying in depth from 2 to 6 hidden layers, where all layers within a given network share a uniform width selected from {32, 64, 128, 256}. A series of networks are trained and evaluated by mBoost. The selection procedure stops until mBoost identifies the first non-improvable model (Figure 3B).

Figure 3C gives the mean-squared error (MSE) on the training set with mBoost assessment results for each depth-width combination. The architecture with a depth of 3 and a width of 128 was the first one identified as non-improvable by mBoost. Consequently, we select this configuration as our final model. Within the hyper-parameter search space, our method requires only 7 full training runs to identify this satisfactory architecture. In contrast, the 5-fold cross-validation strategy employed by Precily needs 100 complete training procedures. This accelerates the architecture selection process and conserves significant computational resources. Furthermore, our model is more parsimonious, containing fewer hidden layer neurons than the Precily model (Figure 3D).

Next, we compare the performance of our model with that of Precily. We calculate the per-drug correlation coefficients between the predicted and the true IC_{50} values on the test set based on these two models. As shown in Figure 3E, our model achieves predictive performance nearly identical to that of Precily despite using fewer parameters. Moreover, the overall predictive accuracy with $R^2 = 0.77$ of our model (two-sided t test for Pearson's correlation, $n = 7{,}794$ cell line-drug pairs, $p < 2.2 \times 10^{-16}$; Figure 3F) is also on par with that of Precily.[35] In summary, mBoost provides an efficient way for selecting hyper-parameters in DNNs. By obviating the need for an exhaustive search across all parameter combinations, mBoost substantially reduces the computational cost for network optimization.

mBoost enhances confidence and accuracy on predicting diseases from gut microbiome

Now, we evaluate the performance of mBoost in building predictive models for complex diseases from gut microbiome data. We collect 120 cohorts including 20 diseases categorized into 5 types (Figure 4A; methods). For the 16S data, we use only the genus-level data for modeling, while for the metagenomic data, both genus- and species-level data are utilized. For each disease and data type, there are at least two cohorts to enable external cohort validation.

We initially construct single-cohort models by using LASSO (a linear model) and evaluate within-cohort cross-validation performance by using AUC. We then apply mBoost to these models and classify them as "improvable" (I) and "non-improvable" (NI) (Figure S5). We identify a total of 15 improvable models out of 120 cohorts (Figure S6A). As expected, the performance of the improvable models is significantly increased after switching to more complex models, such as RF (Figures S6B and S6C), whereas non-improvable models show no substantial improvement (Figure S5). These results affirm the accuracy and validity of our classification.

Next, we focus on feature quality in these cohorts. Of the 120 cohorts, 38 are classified as having redundant features ($p_r > 0.05$, denoted by R; Figure S7A). After feature selection using four different methods (see methods), we observe a significant increase in the internal validation performance (Figure S8). In contrast, no improvement could be achieved for cohorts with non-redundant features (NR) using any of these feature selection methods. This indicates the power of mBoost in identifying redundant features. Further applying the feature validity test,

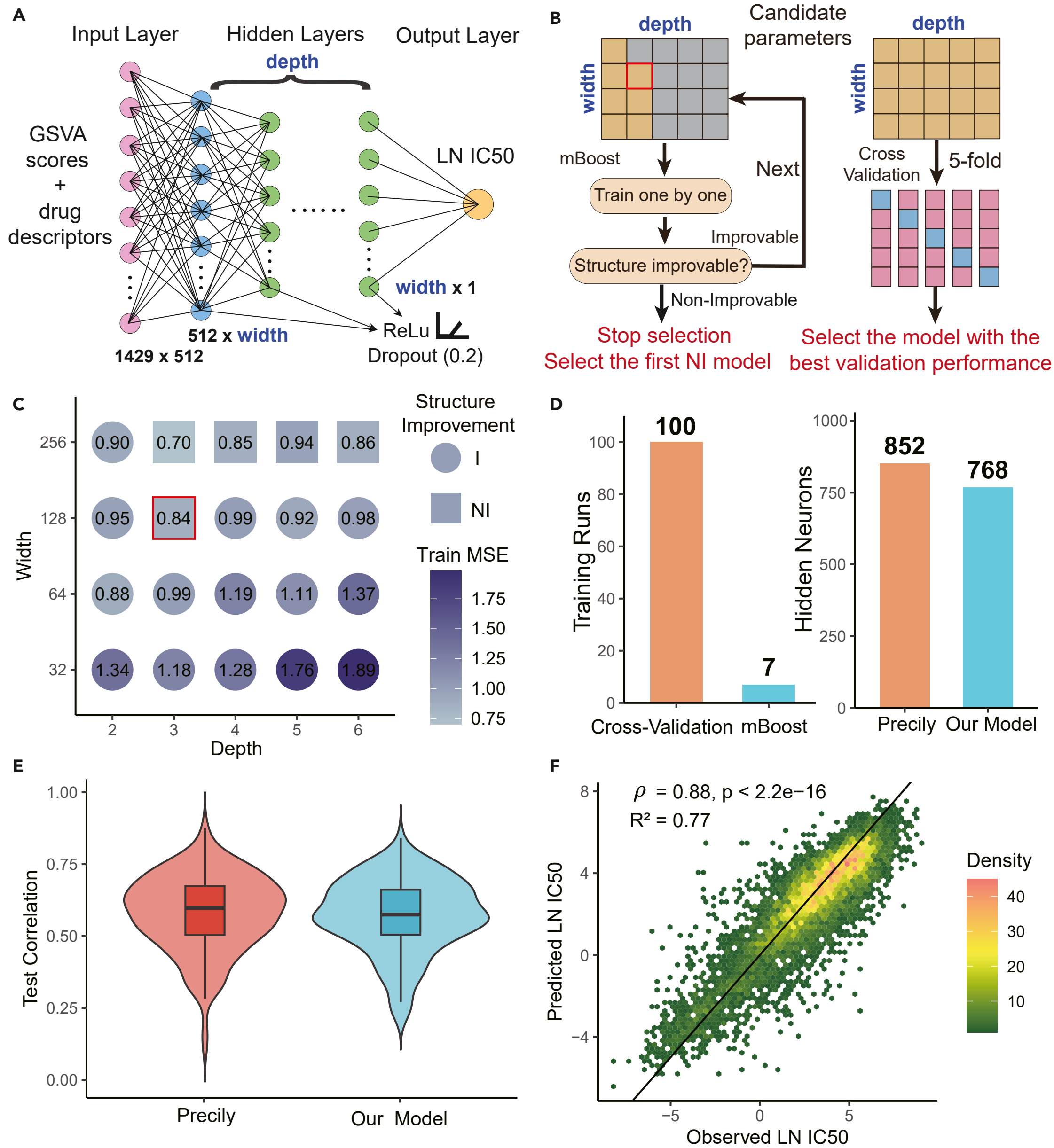

Figure 3. Selecting optimal neural network for predicting drug sensitivity with guidance from mBoost

(A) Neural network architecture for drug sensitivity prediction. The model takes the GSVA scores and drug descriptors as input (1,429 dimensions) to predict drug response, measured by IC_{50}. The depth of the hidden layers and the width after the second layer are the parameters to be determined.

(B) Schematic of the process of cross-validation and mBoost for hyper-parameter selection.

(C) The training mean squared error (MSE) obtained using neural networks with different depths and widths, together with their corresponding structure improvement evaluation outcomes. The selected model is highlighted with a red box.

(D) Left: the number of training epochs required for mBoost and cross-validation-based methods over the candidate hyper-parameter space to find the best model. Right: the number of neurons in the hidden layer in Precily and our selected model.

(E) Comparison of performance (measured by correlation) between Precily and our selected model on the test set.

(F) Scatterplot demonstrating the performance of our model across all cell line-drug pairs in the test data. Statistical significance was assessed using a two-sided *t* test for Pearson's correlation (n = 7,794 cell line-drug pairs, $p < 2.2 \times 10^{-16}$).

we identify 37–86 cohorts that are classified as having valid features (VFs) by using feature selection methods including KS, AD, and Wilcoxon (Figures S7B and S9A). In particular, Wilcoxon classifies more cohorts into the VF group, whereas the other two identify less and only a subset of those identified by Wilcoxon (Figure S9B). We thus use Wilcoxon for feature selection in subsequent analyses. Focusing on 38 cohorts in the R group, we identify 11 among them as having VFs (Figure S7C) and observe significantly higher improvements in performance after feature selection for cohorts with VFs than for cohorts with invalid

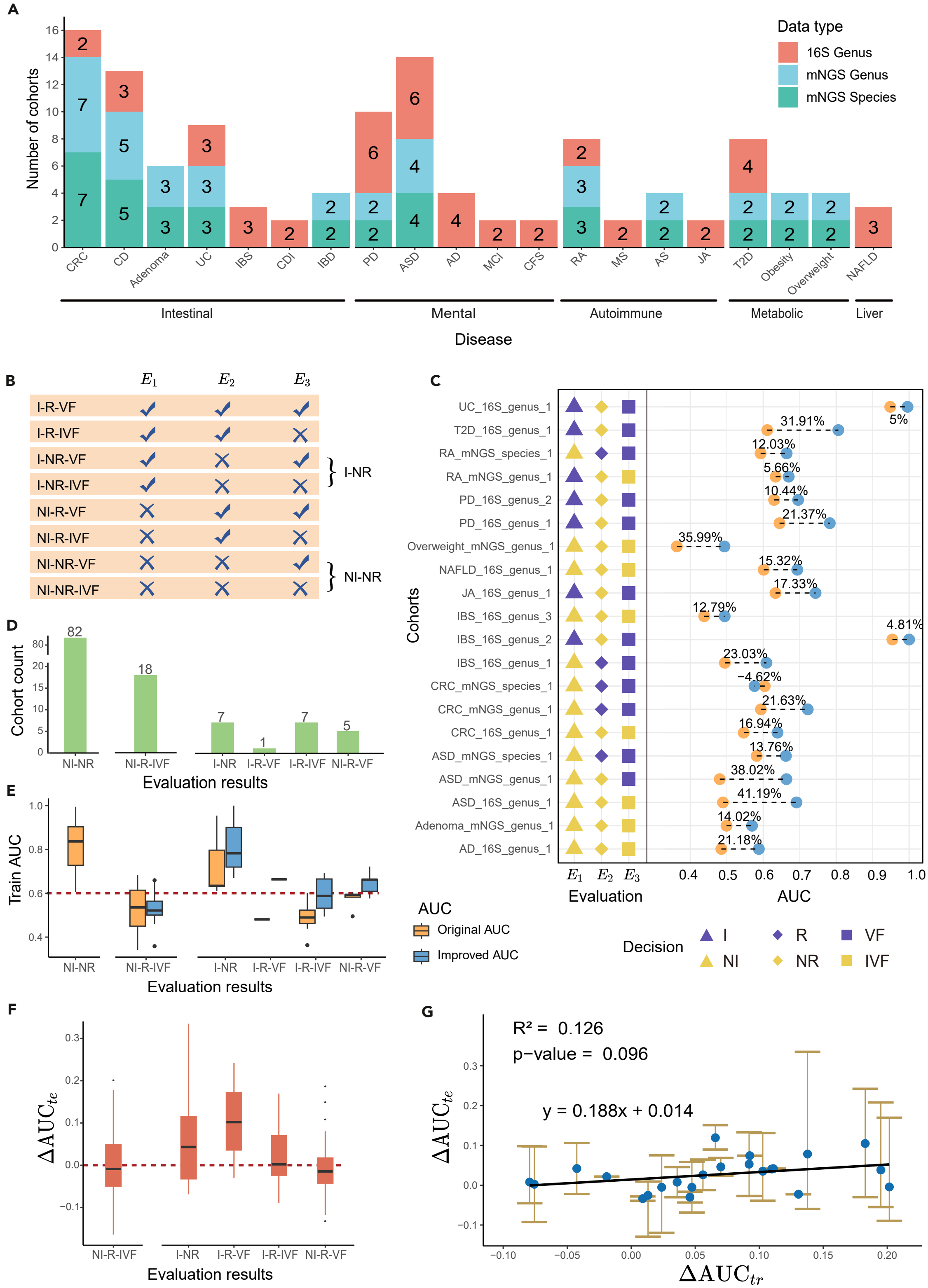

Figure 4. The improvement of the internal training AUC for the microbiome cohorts following the guidance of mBoost

(A) A total of 120 cohorts with diseases categorized into 5 different types.

(B) All possible evaluation results for a machine learning model, where E_1 is for the model quality, E_2 is for feature redundancy, and E_3 is for feature validity (Figure 1). There are eight possible results.

(legend continued on next page)

features (IVFs) (Figure S7D). This indicates the power of mBoost on identifying valid features.

We now follow the suggestions from mBoost to improve the model performance. Given the three evaluations, namely structure improvement (decision: I or NI), feature redundancy (decision: R or NR), and feature validity (decision: VF or IVF), there will be eight possible evaluation results for each model; six of them indicate that the model is improvable (Figure 4B). Among the 120 microbiome cohorts, 20 are deemed to be improvable. Among the non-improvable cohorts, 82 and 18 belong to the NI-NR and NI-R-IVF categories, respectively (Figure 4C). That is, the linear model is good enough for 83.3% cohorts, which is further evidenced by the fact that applying neither feature selection nor complex ML algorithms increases their AUCs. Among the improvable cohorts, 7, 1, 7, and 5 belong to the I-NR, I-R-VF, I-R-IVF, and NI-R-VF groups, respectively (Figure 4C). As expected, we obtain significantly higher AUCs in each of these groups (Figure 4D). Consistently, all but one cohort shows an increase in the AUCs ranging from 5% to 41% depending on the cohorts (Figure 4E). The only exception is a cohort of CRC belonging to the NI-R-VF group, showing a decreased AUC, which indicates the overfitting of the initial model. Interestingly, the improvement gained from mBoost appears to be independent of the original model performance, i.e., any model, regardless of its current accuracy, can be enhanced if it is deemed improvable (Figure 4D), consistent with our previous result that model improvability is independent of model accuracy (Figure 2B).

We next evaluate whether models with improved training AUC can result in improved test AUCs when applied to external datasets (e.g., different cohorts of the same diseases), as the latter is often considered as the gold standard for model generalization. We observe significantly higher test AUCs on some of improved models compared with the original models (Figure 4F). However, there is no significant association between the improved training and test AUCs (linear regression slope test, $n = 60$, $p = 0.096$; Figures 4G and S10), consistent with results shown in the right of Figure S7D. This is further supported by the large variation in improvements of test AUC based on the same improved model (Figure 4G), i.e., the same model has different test AUCs when applied to different datasets. These results motivate us to further explore key factors governing the generalization of the trained models.

Evaluating whether the trained model is applicable to new data

The observation that the improved models on the training datasets do not always give improved test performance on external cohorts (Figures S6D and S7D; Figures 4E, 4F, and S10) strongly indicates that model generalization can be influenced by additional factors.

One critical factor is the similarity between the training and test datasets. In practice, datasets from different sources may exhibit significant discrepancies due to variations in data collection methods and origins.[25] It is, thus, essential to assess the similarity between the training datasets and the test datasets when applying a trained model. Here, we propose a statistical framework, coverage ratio (CR), for assessing dataset similarity (Figure 5A; see methods). The CR ranges in [0,1], and a larger value indicates a higher similarity between datasets. To validate our method, we calculate the correlation between CR and the AUC changes between the training and test dataset and find a significant negative correlation between them (Pearson's correlation $\rho = -0.25$, p value $= 4.24 \times 10^{-6}$; Figure 5B). This is expected because the higher the CR value, the smaller the difference between the training and test datasets.

We further illustrate the use of the CR on two typical scenarios, represented by the cohorts of CRC and Parkinson's disease (PD). The first scenario, consisting of seven CRC cohorts, shows high similarity across cohorts (CR = 0.76, SD = 0.14; Figure 5C). As expected, when training models using individual cohorts and applying them to other cohorts, we find only a slight performance decrease on the latter (ΔAUC = Training AUC-Test AUC = 0.03, SD = 0.12; Figure 5D). In contrast, the second scenario, consisting of six PD cohorts, shows substantially higher cross-cohort heterogeneity (CR = 0.30, SD = 0.08; Figure 5E). Consequently, we find a significantly larger decrease of test performance on external cohorts (ΔAUC = 0.12, SD = 0.29; Figure 5F).

These results align with the physiological conditions of these diseases. Gut microbiome dysbiosis has been directly linked to CRC progression and has been consistently observed across various cohorts, including the enrichment of *Peptostreptococcus stomati*, *Fusobacterium nucleatum*, and *Gemella morbillorum* in patients.[36] In contrast, PD as a neurological condition shows low consistency in gut microbiome alterations overall.[37] Our previous analysis suggests that high-performance cross-cohort machine learning models for CRC can be built with relatively small sample sizes, while those for PD require a significantly higher number of samples from multiple cohorts to accurately capture disease-relevant signals.[2]

In summary, the CR effectively captures the similarity between the test set and the training set, thereby predicting the model performance on the test set.

DISCUSSION

Building predictive models with satisfactory performance relies on the comprehensive and mechanistic understanding of the

(C) The distribution of the number of cohorts on six evaluation results.
(D) The training AUC of cohorts with different evaluation results before and after the improvement. The original AUC is the AUC of linear model with LASSO, and the improved AUC is the AUC of RF taking input features selected by Wilcoxon.
(E) The training AUC before and after improvement with the corresponding increased ratios for all improvable cohorts. For the cohorts with redundant features, only those with valid features are presented.
(F) The improved test AUC, ΔAUC_{te}, on cohorts with different evaluation results from mBoost, where ΔAUC_{te} is the difference between AUCs before and after improving the model under the guidance from mBoost.
(G) The relationship between ΔAUC_{te} and ΔAUC_{tr}, where ΔAUC_{te} is defined as in (F), and ΔAUC_{tr} is the improved AUC on training data. The association in (G) was assessed using a linear regression model; the reported p value is from a two-sided t test for the slope ($n = 60$ and $p = 0.096$).

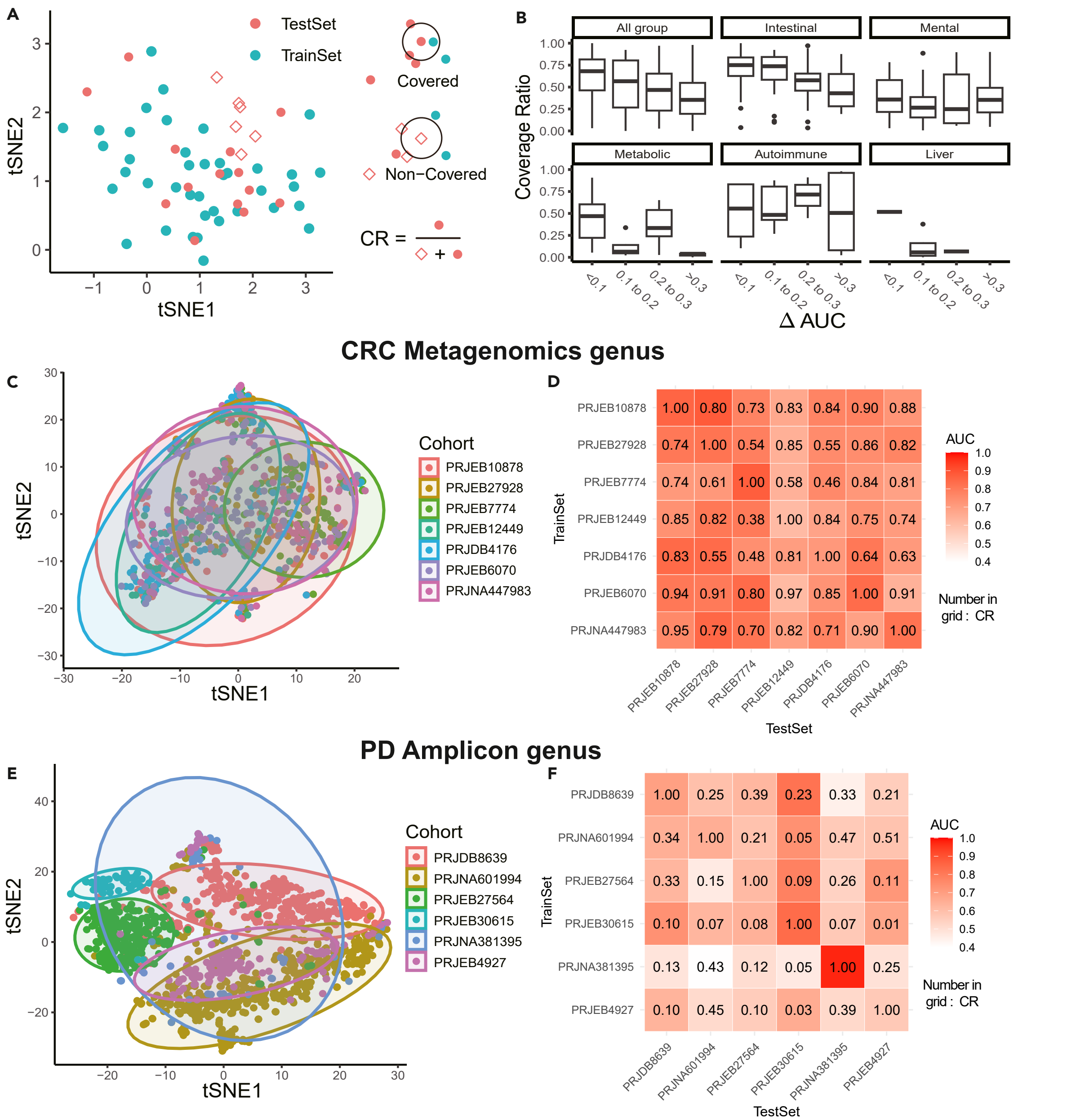

Figure 5. The relationship between the CR and ΔAUC = Training AUC-Test AUC

(A) An illustration of the definition of CR based on low-dimensional representation of the data from t-SNE.

(B) The relationship between Δ AUC and CR across cohorts of different disease types.

(C) t-SNE plot of all cohorts of CRC.

(D) A heatmap depicting the performance of ML models when these CRC cohorts are used as training and test sets for each other. The AUCs are presented in color, while CR values are presented in the grid.

(E) t-SNE plot of amplicon genus-level data of the disease PD.

(F) A heatmap depicting the performance of ML models when these PD cohorts are used as training and test sets for each other. The AUCs are presented in color, while CR values are presented in the grid.

modeling process at both training and test stages. Here, we present mBoost, a statistical framework, to comprehensively assess ML models, identify critical pitfalls, and provide performance enhancing guidance. mBoost employs four statistical measurements, covering three essential steps of building predictive models—including evaluations on model structure, feature quality, and model generalizability to new datasets—providing step-by-step guidance for model improvement and optimization. Validated on both simulated and real-world datasets, mBoost shows promising performance on guiding the optimization of the ML predictive models. Although its performance is shown by using cohorts with microbiome data, mBoost

is widely applicable to predictive models taking other omics data as input, such as RNA-seq, proteomics, DNA methylation, and so on. Thus, we believe that mBoost will serve as an important tool for evaluating ML predictive models.

mBoost can be distinguished from existing methods in the following aspects. First, existing methods for goodness-of-fit test are designed for specific models, such as linear,[38,39] single-index,[40] and heteroscedastic regression models,[41,42] limiting their broad applications. In other words, there is a lack of methods for many other models. Differently, mBoost is assumption-free and model-free and, thus, broadly applicable. The advantage of mBoost comes from its new perspective on goodness-of-fit test that the distance between the working model and true model is equivalent to the distance between the distributions of data from these two models. This enables us to check model optimality without any assumptions on data or model. Importantly, we show that model accuracy is independent of model optimality. Second, mBoost evaluates the quality of features from their number and validity. By identifying low-quality features, mBoost provides guidance on improving model performance by performing feature selection or using alternative feature selection methods.

We show that test accuracy is impacted by the similarity between training data and new data and propose using the CR to measure the similarity. In real examples, the CR effectively captures the similarity between the test and training sets, thereby predicting model performance on the test set. Now, the CR is defined on the two-dimensional representation from t-SNE (t-distributed stochastic neighbor embedding) as it projects the original data from a high-dimensional space into a lower-dimensional space while preserving local similarities in the data. In other words, t-SNE maintains the inherent structure and relationships in the high-dimensional data that is crucial for data representation. In fact, we also try to define the CR based on the low-dimensional representation from other methods, e.g., principal component analysis (PCA) and PCoA (Figure S11). We observe that due to the complexity of microbiome data, the first few principal components (PCs) often do not capture the main information in the data and, thus, fail to represent the entire dataset adequately.

Despite its satisfactory performance on both simulations and examples, mBoost also has some limitations. First, the evaluation of structure improvability is based on a non-parametric statistical test, which requires a sufficient sample size to ensure a high statistical power, especially when there are a large number of features. Second, because the evaluation on feature redundancy and validity relies on the permutation test, applying mBoost to computationally expensive models (such as DNNs) or high-dimensional datasets can be time consuming. Additionally, mBoost provides only a framework to evaluate the feature quality, but it cannot provide clues on how to choose feature selection methods before applying them to data. Third, the CR requires access to the original data of the training set, as it needs to project both the training and test sets onto the same two-dimensional plane. In practice, this may pose potential privacy concerns. To address this issue, future research should explore other methods that involve privacy-preserving techniques, such as data anonymization or federated learning, to ensure that sensitive information is not disclosed, while retaining the ability to assess similarity between datasets. Finally, mBoost currently is designed for evaluating models taking numerical inputs, and its extension to sequence datasets such as DNA sequencing (DNA-seq), proteomics, and RNA-seq will be promising.

METHODS

Overview of mBoost

Model complexity is primarily determined by two aspects: (1) the number of variables, where fewer variables result in a simpler model, and (2) the model structure. For instance, with two variables, one could use a straightforward linear model or a more intricate model that includes interactions and nonlinear relationships between variables. In addition, the quality of covariates (features) included in a model has a high impact on the performance of the model. Low-quality features often result in low performance. Finally, at model application or test stage, the trained model is applied to new datasets. However, one often ignores whether the model is suitable to new datasets. Thus, we propose mBoost as a statistical framework for model evaluation at the training stage and test stage (Figure 1). Based on these evaluations, modeling recommendations are provided. Specifically, we first evaluate whether the model structure is good enough (E_1) and then evaluate the quality of predictors from their numbers (E_2) and validity as predictors for the phenotype (E_3). Finally, we evaluate whether the trained model is applicable to new datasets (E_4). The details of these evaluations are given below.

Evaluation on model structure

In this step, we should test whether the model is good enough, that is, we should perform a goodness-of-fit test for the working model. To begin, we assume the true model between predictors **X** and phenotype Y as

$$Y = c + f(\mathbf{X}) + \epsilon, \text{with } E\epsilon = 0 \quad \text{and} \quad E\epsilon^2 < +\infty, \quad \text{(Equation 1)}$$

where **X** is a vector in $\mathbb{R}^p$ with $p \geq 1$. To make the constant c estimable, we need the identifiability assumption, $Ef(\mathbf{X}) = 0$.

The goal of statistical modeling is to find a parametric model $g(\mathbf{X}|\theta)$ to approximate the unknown function $f(\mathbf{X})$ in Equation 1, where the parameter θ is unknown and will be estimated from observations $\{(\mathbf{x}_i, y_i): i = 1, 2, \ldots, n\}$. If the distribution of ϵ is assumed to be normal, which is common in applied statistics, θ can be estimated by maximizing the likelihood function or Bayesian method. If one does not put any assumption on the distribution of ϵ, θ can be estimated by the least square method or estimating equation approach.

Assuming a working model $g(\mathbf{X}|\theta)$ is proposed for observations from Equation 1, to what extent can we believe in the model $g(\mathbf{X}|\theta_n)$, where θ_n is the estimator of θ. In other words, is there any chance for us to improve our working model? In statistical literature, methods for goodness-of-fit test are proposed for special models, such as linear models,[38,39] single-index models,[40] and heteroscedastic regression models.[41,42] Here, we reframe this problem as a two-sample distribution comparison, making the approach broadly applicable to various models.

From goodness-of-fit test to two-sample distribution comparison

Our idea is that if the working model is good enough, observations from the working model should be very similar to the true

CellPress
OPEN ACCESS

observations (observations from the true model). To make it clear, according to Equation 1, we have the following equation:

$$Y = c + g(\mathbf{X}|\theta) + \eta, \text{with} \quad \eta = f(\mathbf{X}) - g(\mathbf{X}|\theta) + \epsilon, \text{and } Eg(\mathbf{X}|\theta) = 0 \text{ for any } \theta. \quad \text{(Equation 2)}$$

The estimate of θ in Equation 2 is denoted as $\widehat{\theta}_n$. For the case that Y is continuous, we get estimates of η denoted by $\widehat{\eta} = \{\widehat{\eta}_i : i = 1,2,\ldots,n\}$ corresponding to $\{(\mathbf{x}_i,y_i):i = 1,2, \ldots, n\}$ with $\widehat{\eta}_i = y_i - g(\mathbf{x}_i|\widehat{\theta}_n)$. Let $\eta^* = \{\eta_i^* : i = 1,2,\ldots,n\}$ be an i.i.d. bootstrap sample from $\widehat{\eta}$, then $Y^* = g(\mathbf{X}|\widehat{\theta}_n) + \eta^*$ is an i.i.d. predicted sample of Y. If Y is a categorical variable, we can obtain Y^* through a parametric bootstrap. With these notations, we have the following lemma, whose proof is direct and, thus, omitted:

Lemma 1: assuming that $\widehat{\theta}_n \to \theta_0$ almost surely as $n\to\infty$, we have $P\{(\mathbf{X},Y^*) \overset{d}{=} (\mathbf{X},Y)\} = 1$, if and only if the true model $f(\mathbf{X})$ belongs to the parametric model $g(\mathbf{X}|\theta)$, i.e., there exists some θ_0 such that $f(\mathbf{X}) = g(\mathbf{X}|\theta_0)$, where $(\mathbf{X},Y^*) \overset{d}{=} (\mathbf{X},Y)$ means that $(\mathbf{X},Y^*)$ and $(\mathbf{X},Y)$ have the same distribution.

Traditionally, to make statistical inference on goodness-of-fit, one needs to specify the alternative hypothesis or estimate f in a larger functional space[43]; thus, it is not applicable to many cases. Differently, lemma 1 bridges the original problem of measuring the goodness of the working model to the problem of two-sample distribution comparison, i.e., measuring the distance between the distribution of observations $\{(\mathbf{x}_i,y_i):i = 1,2, \ldots, n\}$ from the true model and the distribution of bootstrap samples $\{(\mathbf{x}_i,y_i^*) : i = 1,2,\ldots,n\}$ from the working model. Thus, our idea is more efficient and applicable to many more cases.

MAC statistic for two-sample distribution comparison

The discussion above motivates us to consider the following problem. Given the observations $(\mathbf{X},\mathbf{Y}) \in \mathbb{R}^p \times \mathbb{R}^q, \{(\mathbf{x}_i,\mathbf{y}_i) : i = 1,2,\ldots,n\}$ and $(\mathbf{X},\mathbf{Y}^*) \in \mathbb{R}^p \times \mathbb{R}^q, \{(\mathbf{x}_i,\mathbf{y}_i^*) : i = 1,2,\ldots,n\}$, where $p \geq 1$ and $q \geq 1$ are two positive integers, we want to test the null hypothesis $(\mathbf{X},\mathbf{Y}^*) \overset{d}{=} (\mathbf{X},\mathbf{Y})$.

To define our statistic, some notations are needed. Let $L_i = (\mathbf{w}_i,\mathbf{v}_i), i = 1,2, \ldots, k$, be k points in $\mathbb{R}^p \times \mathbb{R}^q$, where $\mathbf{w}_i \in \mathbb{R}^p$ and $\mathbf{v}_i \in \mathbb{R}^q$. We define

$$A_{L_i,L_j} = \{(\mathbf{x},\mathbf{y}) \in \mathbb{R}^p \times \mathbb{R}^q : d(\mathbf{x},\mathbf{w}_i) \leq d(\mathbf{w}_i,\mathbf{w}_j)\},$$

and

$$B_{L_i,L_j} = \{(\mathbf{x},\mathbf{y}) \in \mathbb{R}^p \times \mathbb{R}^q : d(\mathbf{y},\mathbf{v}_i) \leq d(\mathbf{v}_i,\mathbf{v}_j)\}.$$

Furthermore, we define

$$A_{11} = A_{L_i,L_j} \cap B_{L_i,L_j}, A_{12} = A_{L_i,L_j} \cap B^C_{L_i,L_j},$$

$$A_{21} = A^C_{L_i,L_j} \cap B_{L_i,L_j}, A_{22} = A^C_{L_i,L_j} \cap B^C_{L_i,L_j}.$$

Let

$$P_{ij} = \sum_{k=1}^{n} \mathbb{I}((\mathbf{x}_k,\mathbf{y}_k) \in A_{ij}), Q_{ij} = \sum_{k=1}^{n} \mathbb{I}((\mathbf{x}_k,\mathbf{y}_k^*) \in A_{ij}),$$

and

$$R_{ij} = P_{ij} + Q_{ij},$$

for $i = 1,2; j = 1,2$. The local test statistic at (L_i,L_j) is defined as follows:

$$S(L_i,L_j) = \sum_{k=1}^{2}\sum_{l=1}^{2} \frac{(R_{kl} - Q_{kl})^2}{R_{kl}}. \quad \text{(Equation 3)}$$

If $R_{kl} = 0$, we define the corresponding term in the above formula as 0. It can be shown that $S(L_i, L_j)\to\chi_3^2$, as $n\to\infty$ (see lemma S1 in supplemental information), and, thus, $S(L_i,L_j)$ is called the adjusted chi-squared statistic. Define $D = (\mathbf{X},Y)$ and $D^* = (\mathbf{X},Y^*)$. Now, the MAC test statistic is defined as the maximum of all local statistics:

$$\text{MAC}(D,D^*) = \max_{1\leq i\leq j\leq k} S(L_i,L_j).$$

We reject $H_0 : D \overset{d}{=} D^*$ if $\text{MAC}(D,D^*) > c_0$, where c_0 is a positive threshold. MAC is a consistent test statistic,[30] i.e., as long as the sample size is large enough, MAC can detect any difference in these two distributions.

To establish the consistency of the MAC statistic in our algorithm, we define the population version of the local test statistic at (L_i,L_j) as follows:

$$S^p(L_i,L_j) = n\sum_{k=1}^{2}\sum_{l=1}^{2} \frac{(p_{kl} - q_{kl})^2}{r_{kl}}, \quad \text{(Equation 4)}$$

where $p_{kl} = P_D(A_{kl})$, $q_{kl} = P_{D^*}(A_{kl})$, and $r_{kl} = p_{kl} + q_{kl}$. Furthermore, the population version of MAC statistic is given by

$$\text{MAC}^p(D,D^*) = \max_{1\leq i<j\leq k} S^p(L_i,L_j). \quad \text{(Equation 5)}$$

The consistency of MAC is established in theorem 1.

Theorem 1: following the above notations and assuming that $r_{ij} \geq \eta > 0$ for any i and j, then for any $\epsilon > 0$, let $\delta = \frac{4n\epsilon}{\eta}\left(\frac{1}{\eta} + 2\right)$, we have

$$P(|MAC(D,D^*) - MAC^p(D,D^*)| > \delta) < 4k^2 e^{-n\epsilon^2/8}.$$

Furthermore, we have the following: (1) under H_0, $P(MAC(D,D^*) \leq O(\sqrt{n\log k}))\to 1$, as $n\to\infty$ and (2) under H_1, $P(MAC(D,D^*) > O(n))\to 1$, as $n\to\infty$.

The upper bound of MAC under the null hypothesis does not depend on where we define the local statistics, i.e., the choice of $\mathbf{L}_i$, but it depends only on the number of these locations. This property is key to our algorithm. The theoretical proof of theorem 1 is detailed in supplemental information.

The algorithm

Now, we can evaluate the working model by comparing distributions between the predicted samples from the working model and the true observations from the true model. According to this principle, we propose a new algorithm for model evaluation. The algorithm goes in two steps: (1) estimating the discrepancy between the true model and the working model by MAC statistic and (2) getting the p value for this discrepancy. In step 1, the discrepancy is estimated by comparing the distribution of observations from the true model and that of predicted observations obtained from the working model via bootstrapping. In step 2, the p value is obtained through simulation. The algorithm is summarized in Algorithm 1. The key ingredient in our algorithm

Algorithm 1. Evaluation on model structure

Input: observations: $\{(x_i,y_i):i = 1,2, \ldots,n\}$; working model: $g(X|\widehat{\theta}_n)$;
Output: p_s: p value of checking whether the structure of the working model is improvable.

```
for b = 1, …,B do
    for i = 1,2, …,n do
        Generate a Bootstrap sample of y_i, y_i^{*b}, by bootstrapping the residuals or parametric Bootstrap;
    end for
    Compute the maximum adjusted chi-squared (MAC) test statistic, T_b, between observations {(x_i,y_i):i = 1, 2, …,n} and the Bootstrap sample {(x_i, y_i^{*b}) : i = 1,2,…,n};
end for
Estimate the discrepancy between the true and working model as T_B = (1/B) ∑_{b=1}^{B} T_b.
Compute the p value p_s using Equation 6.
```

is that we use MAC statistic, T_B, in Algorithm 1, to measure the discrepancy between the true model and the working model bycomparing the distribution of observations from the true model and that of predicted observations from the working model. This ingredient allows us to give a full characterization of the discrepancy between the true model $f(\mathbf{X})$ and the working model $g(\mathbf{X}|\theta)$. In other words, T_B can be regarded as the distance between the true model and the working model. The empirical null distribution is obtained by comparing distributions between the bootstrap samples $D^b = \left\{\left(x_i, y_i^{*b}\right) : i = 1,2,\ldots,n\right\}, T_b^*$; thus, the p value is given by

$$p_s = \frac{\sum_{b=1}^{B} \mathbb{I}\left(T_B \leq T_b^*\right) + 1}{B+1}, \quad \text{(Equation 6)}$$

and $p_s \leq 0.05$ indicates that the structure of the working model can be improved.

Evaluation on feature redundancy

Next, we evaluate the quality of covariates. First, we check whether there are too many covariates (feature redundancy) in the model, i.e., whether we need to perform feature selection. In fact, feature redundancy refers to the inclusion of noisy variables in the model. Noisy variables are the features independent of the phenotype. When there are too many of these variables, the model tends to be overfitting, and its interpretability decreases.

Motivated by this fact, we propose to evaluate feature redundancy by comparing the performance of the model on predicting the true phenotype with that on predicting a random phenotype. Specifically, given the working model, we first fit the model to the true observations and get the accuracy, A_0. Then, we permute the phenotype and get the permuted accuracy, A_t, by refitting the working model to the permuted observations. If there is no information redundancy, the permuted accuracy should be much lower than the original one. Thus, the p value is defined as follows:

$$p_r = \frac{\sum_{t=1}^{T} \mathbb{I}(A_0 \leq A_t) + 1}{T+1}. \quad \text{(Equation 7)}$$

If $p_r \leq 0.05$, the accuracy of the working model from training with the original dataset is significantly higher than that from training with the shuffled data, indicating that there is no information redundancy and that feature selection is not necessary. Conversely, if $p_r > 0.05$, we conclude that the original dataset exhibits information redundancy and suggest to perform feature selection. The algorithm is summarized in Algorithm 2.

The accuracies A_0 and A_t are measures of the goodness-of-fit to evaluate how well the working model fits the observed data, and their definition depends on the data type of the phenotype Y. When Y is a binary variable, common metrics include accuracy, precision, recall, F1-score, and AUC. If Y is a continuous variable, regression metrics such as R-squared, mean absolute error (MAE), and root-mean-square error (RMSE) are commonly used. One can choose any of these different metrics according to real situations.

Evaluation on feature validity

Evaluating the validity of features equals to testing the null hypothesis that there are no stable features. To do the hypothesis testing, we first randomly split the dataset into two parts, $(\mathbf{X}_1,Y_1)$ and $(\mathbf{X}_2,Y_2)$. Then, we perform feature selection on each part separately and obtain the feature sets F_1 and F_2, respectively. Let S be the size of the intersection of these two feature sets, i.e., $S = |F_1 \cap F_2|$. Next, we shuffle Y_1 and Y_2, obtaining the shuffled versions Y_1^t and Y_2^t for $t = 1,2, \ldots, T$. For each t, we perform feature selection on the shuffled datasets $\left(X_1, Y_1^t\right)$ and

Algorithm 2. Evaluation on feature redundancy

Input: observations: $\{(\mathbf{x}_i,y_i):i = 1,2, \ldots,n\}$; working model: $g(\mathbf{X}|\theta)$;
Output: p_r: p value of checking whether the features are redundant.

```
Fit the working model g(X|θ) with the original data (X,Y) and calculate the corresponding accuracy A_0;
for t = 1, …,T do
    Randomly permute the original labels Y to construct shuffled data (X,Y^t);
    Fit the working model g(X|θ) with the shuffled data (X,Y^t) and calculate the corresponding accuracy A_t;
end for
Compute the p value p_r based on Equation 7.
```

Algorithm 3. Evaluation on feature validity

Input: observations: ($\mathbf{X}$,Y); a feature selection method;
Output: p_v: p value for testing whether features are valid
Randomly split the dataset into two parts, ($\mathbf{X}_1$,Y_1)and ($\mathbf{X}_2$,Y_2).
Perform feature selection on each part separately, obtaining the feature sets F_1 and F_2, respectively.
for $t = 1, \ldots, T$ **do**
  Randomly permute the original labels Y_1 and Y_2 to construct shuffled data $\left(\mathbf{X}_1, Y_1^t\right)$ and $\left(\mathbf{X}_2, Y_2^t\right)$.
  Perform feature selection on each shuffled data separately, obtaining the feature sets F_1^t and F_2^t, respectively.
end for
Compute the p value p_v based on Equation 8.

$\left(X_2, Y_2^t\right)$, yielding the feature sets F_1^t and F_2^t, and then get the size of their intersections S_t. Note that S_t represents the size of stable features when the phenotype Y is unrelated to X and, thus, serves as the null distribution of S. Thus, the p value is given by

$$p_v = \frac{\sum_{t=1}^{T} \mathbb{I}(S \leq S_t) + 1}{T+1}, \quad \text{(Equation 8)}$$

and $p_v \leq 0.05$ indicates that there are stable features. Otherwise, the selected features are invalid, and we need to switch to another feature selection method. The algorithm is summarized in Algorithm 3. If the selected features are invalid after trying several different feature selection methods, we may conclude that $\mathbf{X}$ and Y are independent in this dataset. In other words, the phenotype Y cannot be predicted by $\mathbf{X}$.

Similarity between training and test data

We propose an index called CR to measure the similarity between the training data and test data. First, we combine the predictors $\mathbf{X}$ from the training set and $\mathbf{X}'$ from the test set and then use t-SNE to obtain their two-dimensional embeddings. In the resulting two-dimensional plot, the CR is defined as the proportion of points in the test set that are "covered" by points in the training set. Coverage here refers to whether a neighborhood, e.g., k-nearest neighbors, of a test point x' contain points from the training set. If the CR is greater than 0.5, we conclude that the model trained on the training set is suitable for the test set; otherwise, it suggests a significant difference between these two datasets, potentially leading to poor test performance. In practice, we choose $k = \lfloor 0.02{*}n \rfloor + 1$, where n is the number of samples in the test set. The algorithm is summarized in Algorithm 4.

Simulation

Example 1: The variable selection capability of Lasso

We illustrate through a simple simulation that the feature selection capability of LASSO decreases as the number of noisy variables increases. Assuming there are p_0+p_1 variables, among them, p_0 variables are relevant to the response Y. Specifically, we generated the data using the model, $Y = X\beta + e$, where

$$X = (x_{ij})_{n\times(p_0+p_1)}, x_{ij} \sim N(0,1), \beta = \left(\beta_1, \ldots, \beta_{p_0}, \ldots, \beta_{p_0+p_1}\right)', \beta_i = 1 \quad \text{for} \quad i = 1, \ldots, p_0,$$

and $\beta_j = 0$ for $j = p_0 + 1, \ldots, p_0 + p_1$. $e = (e_i)_{n\times 1}, e_i \sim N(0,2)$ is the Gaussian noise.

We set $n = 200$, $p_0 = 20$, and $p_1 = 50,100, \ldots, 500$ in the simulation. We fitted the data using LASSO and calculated the number of true positives (TPs), i.e., the number of true features selected by LASSO. The regularization parameter λ in LASSO is selected using cross-validation. In each setting, 100 independent repetitions were performed.

Example 2: Polynomial regression

In this simulation, we assume that the data follow a polynomial model:

$$f(x) = 10(x - 0.2)^2 + \varepsilon,$$

where $x \sim U[0,1]$ and $\varepsilon \sim N(0,1)$. We generated 200 observations from this model and fitted the data using both linear and quadratic regression models.

Example 3: Crescent-shaped classification

In this example, observations are generated from two clusters: observations in class 0 follow the model

Algorithm 4. Evaluation on data similarity

Input: the predictors from training and test set: $\mathbf{X} = \{\mathbf{x}_1, \ldots, \mathbf{x}_n\}, \mathbf{X}' = \{\mathbf{x}_1', \ldots, \mathbf{x}_m'\}$;
Output: CR: measurement on similarity between training set $\mathbf{X}$ and test set $\mathbf{X}'$.
Combine $\mathbf{X}$ and $\mathbf{X}'$ together and conduct dimension reduction using t-SNE.
Set C = 0, initialize the number of covered points in $\mathbf{X}'$.
for each $\mathbf{x}'$ in $\mathbf{X}'$ **do**
  Let $U_k(\mathbf{x}')$ be the k-nearest neighbors of $\mathbf{x}'$ in the t-SNE plot
  $C(\mathbf{x}') = \{\mathbf{x} \in \mathbf{X}, \mathbf{x} \in U_k(\mathbf{x}')\}$
  if $|C(\mathbf{x}')| > 0$ **then**
    C=C+1
  end if
end for
CR = C/m

$$\begin{cases} x_1 = 5\cos(\theta_1) + \varepsilon, \\ y_1 = 10\sin(\theta_1) + \varepsilon, \end{cases}$$

where $\theta_1 \sim U\left[-\frac{\pi}{4}, \frac{3\pi}{4}\right]$, and ε follows a normal distribution $\varepsilon \sim N(0,0.5)$. Observations in class 1 follow the model

$$\begin{cases} x_2 = 5\cos(\theta_2) + 5 + \varepsilon, \\ y_2 = 10\sin(\theta_2) - 5 + \varepsilon, \end{cases}$$

where $\theta_2 \sim U\left[\frac{3\pi}{4}, \frac{7\pi}{4}\right]$ and $\varepsilon \sim N(0,0.5)$. We generated 200 observations from each of these two classes and randomly took 160 observations as the training set, with the remaining as the test set. We fitted the training set using a linear logistic regression model, a support vector machine with a nonlinear Gaussian kernel, and an RF model. Subsequently, we evaluated their accuracy on the test set.

Example 4: Linear logistic regression

In this example, the dataset (X,Y) follows the model,

$$y_i \text{ follows a binomial distribution, i.e., } y_i \sim \mathcal{B}(1, p_i), \text{ and}$$

$$\ln\left(\frac{p_i}{1 - p_i}\right) = x_i'\beta + \beta_0, \text{with } x_i, \beta \in \mathbb{R}^p$$

where $X = (x_1', x_2', \ldots, x_n')'$ and each dimension of x_i $(i = 1,2, \ldots, n)$ is drawn from an independent normal distribution. Twenty non-zero entries are randomly selected from β, with values independently drawn from a normal distribution, while the remaining $p_1 = p$-20 terms are set to 0. Thus, only the corresponding 20 dimensions of X are relevant to Y, while the rest are noisy variables. 200 observations are generated as the training set and another 40 observations as test set. The number of the noisy variables p_1 ranges from 30 to 100. In each setting, we first fitted the model using logistic regression and then evaluated information redundancy, using mBoost (p_r). Next, we used the LASSO-based logistic regression model to fit the data and evaluated feature redundancy and feature validity in the model by using mBoost (p_r and p_v).

Example 5: Feature selection in a nonlinear problem

In this example, we generated data from the following model:

$$\mathbf{x}_i = (x_{i1}, x_{i2}, \ldots, x_{ip}), x_{i1}, x_{i2} \sim U(0,2), x_{i3}, \ldots, x_{ip} \sim N(0,4), \text{for} \quad i = 1,2,\ldots,n.$$

The label is generated by

$$y_i = \begin{cases} 1, & \text{if } x_{i1}^2 + x_{i2}^2 < \frac{8}{\pi}, \\ 0, & \text{if } x_{i1}^2 + x_{i2}^2 \geq \frac{8}{\pi}. \end{cases}$$

In this simulation, p ranges from 100 to 500, and 200 observations were generated as training data. Another 40 observations were taken as test data in each setting.

Example 6: Negative control

To evaluate the robustness of mBoost and ensure it does not yield spurious results, we conducted negative control experiments using simulated null data. Specifically, we generated a feature matrix $X \in \mathbb{R}^{n \times p}$, with each entry independently drawn from a standard normal distribution, and an independent response vector Y, with each entry independently drawn from a binomial distribution. In this simulation, we set the sample size to $n = 200$ and the number of features to $p = 50$. The results were reported from 100 independent repetitions.

Feature selection methods

Assume the phenotype Y is binary and X is a continuous predictor; we wanted to see whether there exists f, such that

$$\log(P(Y=1|X)\,/\,P(Y=0|X)) = c + f(X).$$

Actually,

$$\frac{P(Y=1|X)}{P(Y=0|X)} = \frac{f(X|Y=1)}{f(X|Y=0)} \times \frac{P(Y=0)}{P(Y=1)},$$

thus, $f(X) = \log\left(\frac{f(X|Y=1)}{f(X|Y=0)}\right)$, and $c = \log\left(\frac{P(Y=0)}{P(Y=1)}\right)$. In other words, to test whether X is independent of Y, i.e., $f(X) = 0$, we only need to test whether conditional distributions $(X|Y = 1)$ and $(X|Y = 0)$ are the same. If we assume they are normal with the same variance, we can just compare their means, i.e., performing t test or Wilcoxon test. KS and AD are used to compare the entire distributions. Finally, LASSO and RF actually are model-based variable selection methods, where LASSO assumes that f is a linear model and RF assumes it to be a nonlinear model.

t test

When the t test[44] is used for feature selection, we transformed the feature selection problem into a two-sample mean test problem. Because the phenotype Y is binary, we divided the observations into two groups according to the label and considered features with different means between the groups. Typically, if the p value is below 0.05, it is considered to have a significant difference between the two classes, and, thus, the covariate is a discriminative feature.

Wilcoxon test

The Wilcoxon test[45] is a non-parametric test used for identifying features that did not meet normality assumptions in t test.

Kolmogorov-Smirnov test

The KS test,[46] which is also non-parametric, is applied to compare the conditional distributions of the feature, i.e., $(X|Y = 1)$ and $(X|Y = 0)$.

Anderson-Darling test

The AD test[47] is a powerful statistical test for two-sample distribution comparison, sensitive to both the overall data distribution and tail deviations, suitable for both small and large samples, and applicable to various types of distributions.

LASSO

LASSO is run by R package glmnet (v.4.1-8). The tuning parameter used in LASSO is selected by cross validation.

Random Forest

Feature selection involves fitting an RF model to the training dataset and selecting the top 30 features based on their importance scores. Feature importance was measured using the mean decreasing accuracy, which quantifies how much the predictive accuracy of the model decreases when a particular feature's values are permuted. This approach identifies features that contribute the most to the model's performance, providing a complementary perspective to the statistical tests. RF is

CellPress
OPEN ACCESS

implemented in R package randomForest (v.4.7–1.1). We used the default parameters provided in this package when running the RF.

Real-data analysis

Data collection and pre-processing

The data used in this study were sourced from previous gut microbiome research, containing whole-genome sequencing (WGS) and 16S amplicon data sequencing profiles (https://github.com/whchenlab/GMModels/tree/main/01_data/feat_meta). This research covered 20 diseases across five major categories, using 120 case-control cohorts with a total of 9,708 samples. Each disease includes two or more cohorts, enabling external validation within the same disease.

Among five disease categories, first is the ''intestinal diseases,'' which include CRC, Crohn disease (CD), adenoma, ulcerative colitis (UC), irritable bowel syndrome (IBS), *Clostridium* infection (CDI), and inflammatory bowel disease (IBD). The second category is ''mental diseases,'' which include PD, ASD, Alzheimer's disease (AD), mild cognitive impairment (MCI), and chronic fatigue syndrome (CFS). The third category is ''autoimmune diseases,'' which include rheumatoid arthritis (RA), multiple sclerosis (MS), ankylosing spondylitis (AS), and juvenile arthritis (JA). The fourth category is ''metabolic diseases,'' which include type 2 diabetes mellitus (T2D), obesity, and overweight. The fifth is ''liver diseases,'' which include non-alcoholic fatty liver disease (NAFLD).

The WGS data were profiled using Metaphlan2,[48] retaining genus-level and species-level data for analysis. The 16S amplicon data were profiled using Qiime2 version 2021.2,[49] with genus-level data retained for analysis. Samples with two or fewer taxa were excluded from further analysis. Low-abundance taxa with a relative abundance of less than 0.001 across all samples were also filtered out. We used the relative abundance data to establish patient stratification models and conduct four evaluations to diagnose these models. We defined a cohort as the gut microbiome data from different patients and the corresponding healthy controls in the same project with the same data type. As a result, we got 120 cohorts including 46 16S genus-level, 37 WGS genus-level, and species-level cohorts.

Modeling procedure

For each cohort, we initially used the linear model with LASSO to establish the predictive model. Then, we used mBoost to evaluate the model and followed the guidance to improve the model. The accuracy of the model was evaluated by the internal cross-validation AUC.

RESOURCE AVAILABILITY

Lead contact

Requests for further information and resources should be directed to and will be fulfilled by the lead contact, Hangjin Jiang (jianghj@zju.edu.cn).

Materials availability

This study did not generate any physical materials.

Data and code availability

- Public gut microbiome data used in this study are available from GMModels at https://github.com/whchenlab/GMModels/tree/main/01_data/feat_meta. The drug-sensitivity data used in the Precily example are available from Zenodo[50] at https://doi.org/10.5281/zenodo.7024834.
- Core scripts, analysis data, and generated figures supporting this study have been deposited at Zenodo at https://doi.org/10.5281/zenodo.20786902 and are formally cited in our previous work.[51] The corresponding GitHub repository is available at https://github.com/Lingning927/Scripts_mBoost.
- The mBoost R package has been deposited at Zenodo at https://doi.org/10.5281/zenodo.20786916 and is formally cited in our previous work.[52] The corresponding GitHub repository is available at https://github.com/Lingning927/mBoost.
- Any additional information required to reanalyze the data reported in this paper is available from the lead contact upon request.

ACKNOWLEDGMENTS

H.J. is partially supported by Jianbing&Lingyan Research and Development Program of Zhejiang Province (2024C03239) and High-level Talent Special Support Program of Zhejiang Province. W.-H.C. is partially supported by Double First-Class Construction Funds for Provincial Departments Affiliated Universities in Hubei Province (5001170159).

AUTHOR CONTRIBUTIONS

Conceptualization, H.J.; methodology, B.S. and H.J.; formal analysis, B.S.; investigation, B.S.; data collection, M.L. and B.S.; writing – original draft, B.S. and M.L.; writing – review & editing, B.S., M.L., X.H., M.C., W.-H.C., and H.J.; supervision, W.-H.C. and H.J.; project administration, W.-H.C. and H.J. All authors have approved the final manuscript.

DECLARATION OF INTERESTS

The authors declare no competing interests.

SUPPLEMENTAL INFORMATION

Supplemental information can be found online at https://doi.org/10.1016/j.patter.2026.101643.

Received: February 16, 2026
Revised: June 24, 2026
Accepted: July 31, 2026
Published: September 3, 2026

REFERENCES

1. Thomas, A.M., Manghi, P., Asnicar, F., Pasolli, E., Armanini, F., Zolfo, M., Beghini, F., Manara, S., Karcher, N., Pozzi, C., et al. (2019). Metagenomic analysis of colorectal cancer datasets identifies cross-cohort microbial diagnostic signatures and a link with choline degradation. Nat. Med. *25*, 667–678. https://doi.org/10.1038/s41591-019-0405-7.
2. Li, M., Liu, J., Zhu, J., Wang, H., Sun, C., Gao, N.L., Zhao, X.M., and Chen, W.H. (2023). Performance of gut microbiome as an independent diagnostic tool for 20 diseases: cross-cohort validation of machine-learning classifiers. Gut Microbes *15*, 2205386. https://doi.org/10.1080/19490976.2023.2205386.
3. DeGroat, W., Abdelhalim, H., Patel, K., Mendhe, D., Zeeshan, S., and Ahmed, Z. (2024). Discovering biomarkers associated and predicting cardiovascular disease with high accuracy using a novel nexus of machine learning techniques for precision medicine. Sci. Rep. *14*, 1. https://doi.org/10.1038/s41598-023-50600-8.
4. Lu, M., Yin, R., and Chen, X.S. (2024). Ensemble methods of rank-based trees for single sample classification with gene expression profiles. J. Transl. Med. *22*, 140. https://doi.org/10.1186/s12967-024-04940-2.
5. Garg, M., Karpinski, M., Matelska, D., Middleton, L., Burren, O.S., Hu, F., Wheeler, E., Smith, K.R., Fabre, M.A., Mitchell, J., et al. (2024). Disease prediction with multi-omics and biomarkers empowers case-control

genetic discoveries in the uk biobank. Nat. Genet. *56*, 1821–1831. https://doi.org/10.1038/s41588-024-01898-1.

6. Smelik, M., Zhao, Y., Li, X., Loscalzo, J., Sysoev, O., Mahmud, F., Mansour Aly, D., and Benson, M. (2024). An interactive atlas of genomic, proteomic, and metabolomic biomarkers promotes the potential of proteins to predict complex diseases. Sci. Rep. *14*, 12710. https://doi.org/10.1038/s41598-024-63399-9.
7. You, J., Guo, Y., Zhang, Y., Kang, J.J., Wang, L.B., Feng, J.F., Cheng, W., and Yu, J.T. (2023). Plasma proteomic profiles predict individual future health risk. Nat. Commun. *14*, 7817. https://doi.org/10.1038/s41467-023-43575-7.
8. Bjerrum, J.T., Wang, Y.L., Seidelin, J.B., and Nielsen, O.H. (2021). Ibd metabonomics predicts phenotype, disease course, and treatment response. EBioMedicine *71*, 103551. https://doi.org/10.1016/j.ebiom.2021.103551.
9. Buergel, T., Steinfeldt, J., Ruyoga, G., Pietzner, M., Bizzarri, D., Vojinovic, D., Upmeier zu Belzen, J., Loock, L., Kittner, P., Christmann, L., et al. (2022). Metabolomic profiles predict individual multidisease outcomes. Nat. Med. *28*, 2309–2320. https://doi.org/10.1038/s41591-022-01980-3.
10. Zuccato, J.A., Mamatjan, Y., Nassiri, F., Ajisebutu, A., Liu, J.C., Muazzam, A., Singh, O., Zhang, W., Voisin, M., Mirhadi, S., et al. (2024). Prediction of brain metastasis development with dna methylation signatures. Nat. Med. 1–10.
11. de Vos, W.M., Tilg, H., Van Hul, M., and Cani, P.D. (2022). Gut microbiome and health: mechanistic insights. Gut *71*, 1020–1032. https://doi.org/10.1136/gutjnl-2021-326789.
12. Wu, G., Xu, T., Zhao, N., Lam, Y.Y., Ding, X., Wei, D., Fan, J., Shi, Y., Li, X., Li, M., et al. (2024). A core microbiome signature as an indicator of health. Cell *187*, 6550–6565.e11. https://doi.org/10.1016/j.cell.2024.09.019.
13. Yachida, S., Mizutani, S., Shiroma, H., Shiba, S., Nakajima, T., Sakamoto, T., Watanabe, H., Masuda, K., Nishimoto, Y., Kubo, M., et al. (2019). Metagenomic and metabolomic analyses reveal distinct stage-specific phenotypes of the gut microbiota in colorectal cancer. Nat. Med. *25*, 968–976. https://doi.org/10.1038/s41591-019-0458-7.
14. Oh, T.G., Kim, S.M., Caussy, C., Fu, T., Guo, J., Bassirian, S., Singh, S., Madamba, E.V., Bettencourt, R., Richards, L., et al. (2020). A universal gut-microbiome-derived signature predicts cirrhosis. Cell Metab. *32*, 888–901. https://doi.org/10.1016/j.cmet.2020.10.015.
15. Wan, Y., Zuo, T., Xu, Z., Zhang, F., Zhan, H., Chan, D., Leung, T.F., Yeoh, Y.K., Chan, F.K.L., Chan, R., and Ng, S.C. (2022). Underdevelopment of the gut microbiota and bacteria species as non-invasive markers of prediction in children with autism spectrum disorder. Gut *71*, 910–918. https://doi.org/10.1136/gutjnl-2020-324015.
16. Sharon, G., Cruz, N.J., Kang, D.W., Gandal, M.J., Wang, B., Kim, Y.M., Zink, E.M., Casey, C.P., Taylor, B.C., Lane, C.J., et al. (2019). Human gut microbiota from autism spectrum disorder promote behavioral symptoms in mice. Cell *177*, 1600–1618.e17. https://doi.org/10.1016/j.cell.2019.05.004.
17. Qin, Y., Havulinna, A.S., Liu, Y., Jousilahti, P., Ritchie, S.C., Tokolyi, A., Sanders, J.G., Valsta, L., Brożyńska, M., Zhu, Q., et al. (2022). Combined effects of host genetics and diet on human gut microbiota and incident disease in a single population cohort. Nat. Genet. *54*, 134–142. https://doi.org/10.1038/s41588-021-00991-z.
18. Chen, V., Yang, M., Cui, W., Kim, J.S., Talwalkar, A., and Ma, J. (2024). Applying interpretable machine learning in computational biology—pitfalls, recommendations and opportunities for new developments. Nat. Methods *21*, 1454–1461. https://doi.org/10.1038/s41592-024-02359-7.
19. Lee, S., and Lee, I. (2024). Comprehensive assessment of machine learning methods for diagnosing gastrointestinal diseases through whole metagenome sequencing data. Gut Microbes *16*, 2375679. https://doi.org/10.1080/19490976.2024.2375679.
20. Asnicar, F., Thomas, A.M., Passerini, A., Waldron, L., and Segata, N. (2024). Machine learning for microbiologists. Nat. Rev. Microbiol. *22*, 191–205. https://doi.org/10.1038/s41579-023-00984-1.
21. Fan, Y., and Pedersen, O. (2021). Gut microbiota in human metabolic health and disease. Nat. Rev. Microbiol. *19*, 55–71. https://doi.org/10.1038/s41579-020-0433-9.
22. Wu, G.D., Chen, J., Hoffmann, C., Bittinger, K., Chen, Y.Y., Keilbaugh, S.A., Bewtra, M., Knights, D., Walters, W.A., Knight, R., et al. (2011). Linking long-term dietary patterns with gut microbial enterotypes. Science *334*, 105–108. https://doi.org/10.1126/science.1208344.
23. Zhu, J., Sun, C., Li, M., Hu, G., Zhao, X.M., and Chen, W.H. (2024). Compared to histamine-2 receptor antagonist, proton pump inhibitor induces stronger oral-to-gut microbial transmission and gut microbiome alterations: a randomised controlled trial. Gut *73*, 1087–1097. https://doi.org/10.1136/gutjnl-2023-330168.
24. Wu, S., Jiang, P., Zhao, X.M., and Chen, W.H. (2021). Treatment regimens may compromise gut-microbiome-derived signatures for liver cirrhosis. Cell Metab. *33*, 455–456. https://doi.org/10.1016/j.cmet.2021.02.012.
25. He, Y., Wu, W., Zheng, H.M., Li, P., McDonald, D., Sheng, H.F., Chen, M.X., Chen, Z.H., Ji, G.Y., Zheng, Z.D.X., et al. (2018). Regional variation limits applications of healthy gut microbiome reference ranges and disease models. Nat. Med. *24*, 1532–1535. https://doi.org/10.1038/s41591-018-0164-x.
26. Costea, P.I., Zeller, G., Sunagawa, S., Pelletier, E., Alberti, A., Levenez, F., Tramontano, M., Driessen, M., Hercog, R., Jung, F.E., et al. (2017). Towards standards for human fecal sample processing in metagenomic studies. Nat. Biotechnol. *35*, 1069–1076. https://doi.org/10.1038/nbt.3960.
27. Japkowicz, N., and Shah, M. (2011). Evaluating Learning Algorithms: A Classification Perspective (Cambridge University Press).
28. Tsay, R.S. (1992). Model checking via parametric bootstraps in time series analysis. Appl. Stat. *41*, 1–15. https://doi.org/10.2307/2347612.
29. Gelman, A., Meng, X.L., and Stern, H. (1996). Posterior predictive assessment of model fitness via realized discrepancies. Stat. Sin. *6*, 733–760.
30. Jiang, H., Zhao, X., Ma, R.C.W., and Fan, X. (2022). Consistent screening procedures in high-dimensional binary classification. Stat. Sin. *32*, 109–130. https://doi.org/10.5705/ss.202020.0088.
31. Tibshirani, R. (1996). Regression shrinkage and selection via the lasso. J. Roy. Stat. Soc. B Stat. Methodol. *58*, 267–288. https://doi.org/10.1111/j.2517-6161.1996.tb02080.x.
32. Zou, H., and Hastie, T. (2005). Regularization and variable selection via the elastic net. J. Roy. Stat. Soc. B Stat. Methodol. *67*, 301–320. https://doi.org/10.1111/j.1467-9868.2005.00503.x.
33. Li, H., Zhang, H., and Jiang, H. (2022). Combining power of different methods to detect associations in large data sets. Briefings Bioinf. *23*, bbab488. https://doi.org/10.1093/bib/bbab488.
34. Khaire, U.M., and Dhanalakshmi, R. (2022). Stability of feature selection algorithm: A review. J. King Saud Univ. Comput. Inf. Sci. *34*, 1060–1073. https://doi.org/10.1016/j.jksuci.2019.06.012.
35. Chawla, S., Rockstroh, A., Lehman, M., Ratther, E., Jain, A., Anand, A., Gupta, A., Bhattacharya, N., Poonia, S., Rai, P., et al. (2022). Gene expression based inference of cancer drug sensitivity. Nat. Commun. *13*, 5680. https://doi.org/10.1038/s41467-022-33291-z.
36. Wirbel, J., Pyl, P.T., Kartal, E., Zych, K., Kashani, A., Milanese, A., Fleck, J.S., Voigt, A.Y., Palleja, A., Ponnudurai, R., et al. (2019). Meta-analysis of fecal metagenomes reveals global microbial signatures that are specific for colorectal cancer. Nat. Med. *25*, 679–689. https://doi.org/10.1038/s41591-019-0406-6.
37. Rajput, C., Sarkar, A., Sachan, N., Rawat, N., and Singh, M.P. (2021). Is gut dysbiosis an epicenter of parkinson's disease? Neurochem. Res. *46*, 425–438. https://doi.org/10.1007/s11064-020-03187-9.
38. Stute, W., Thies, S., and Zhu, L.X. (1998). Model checks for regression: an innovation process approach. Ann. Stat. *26*, 1916–1934. https://doi.org/10.1214/aos/1024691363.
39. Stute, W. (1997). Nonparametric Model Checks for Regression (Ann Stat), pp. 613–641. https://doi.org/10.1214/aos/1031833666.
40. Guo, X., Wang, T., and Zhu, L. (2016). Model checking for parametric single-index models: a dimension reduction model-adaptive approach.

J. Roy. Stat. Soc. B Stat. Methodol. *78*, 1013–1035. https://doi.org/10.1111/rssb.12147.

41. Van Keilegom, I., González Manteiga, W., and Sánchez Sellero, C. (2008). Goodness-of-fit tests in parametric regression based on the estimation of the error distribution. TEST *17*, 401–415. https://doi.org/10.1007/s11749-007-0044-z.
42. Neumeyer, N., and Van Keilegom, I. (2010). Estimating the error distribution in nonparametric multiple regression with applications to model testing. J. Multivar. Anal. *101*, 1067–1078. https://doi.org/10.1016/j.jmva.2010.01.007.
43. Fan, J., Zhang, C., and Zhang, J. (2001). Generalized likelihood ratio statistics and wilks phenomenon. Ann. Stat. *29*, 153–193. https://doi.org/10.1214/aos/996986505.
44. Student (1908). The probable error of a mean. Biometrika *6*, 1–25. https://doi.org/10.2307/2331554.
45. Wilcoxon, F. (1945). Individual comparisons by ranking methods. Biometrics Bull. *1*, 80–83. https://doi.org/10.2307/3001968.
46. Smirnov, N. (1948). Table for estimating the goodness of fit of empirical distributions. Ann. Math. Stat. *19*, 279–281. https://doi.org/10.1214/aoms/1177730256.
47. Anderson, T.W., and Darling, D.A. (1952). Asymptotic theory of certain goodness-of-fit criteria based on stochastic processes. Ann. Math. Stat. *23*, 193–212. https://doi.org/10.1214/aoms/1177729437.
48. Segata, N., Waldron, L., Ballarini, A., Narasimhan, V., Jousson, O., and Huttenhower, C. (2012). Metagenomic microbial community profiling using unique clade-specific marker genes. Nat. Methods *9*, 811–814. https://doi.org/10.1038/nmeth.2066.
49. Bolyen, E., Rideout, J.R., Dillon, M.R., Bokulich, N.A., Abnet, C.C., Al-Ghalith, G.A., Alexander, H., Alm, E.J., Arumugam, M., Asnicar, F., et al. (2019). Reproducible, interactive, scalable and extensible microbiome data science using qiime 2. Nat. Biotechnol. *37*, 852–857. https://doi.org/10.1038/s41587-019-0209-9.
50. Chawla, S. (2022). SmritiChawla/Precily: Precily: Release for Zenodo. Zenodo. https://doi.org/10.5281/zenodo.7024834
51. Shen, B., and Jiang, H. (2026). Scripts, analysis data, and generated figures for mBoost: a statistical framework for model evaluation and improvement. Zenodo. https://doi.org/10.5281/zenodo.20786902.
52. Shen, B., and Jiang, H. (2026). mBoost: an R package for model evaluation and improvement, version 1.0.0. Zenodo. https://doi.org/10.5281/zenodo.20786916.

Patterns, Volume *7*

Supplemental information

Identifying low-quality features and suboptimal models to enhance phenotype prediction

Baining Shen, Min Li, Xiaoyue Hu, Ming Chen, Wei-Hua Chen, and Hangjin Jiang

Supplemental Figures and Table

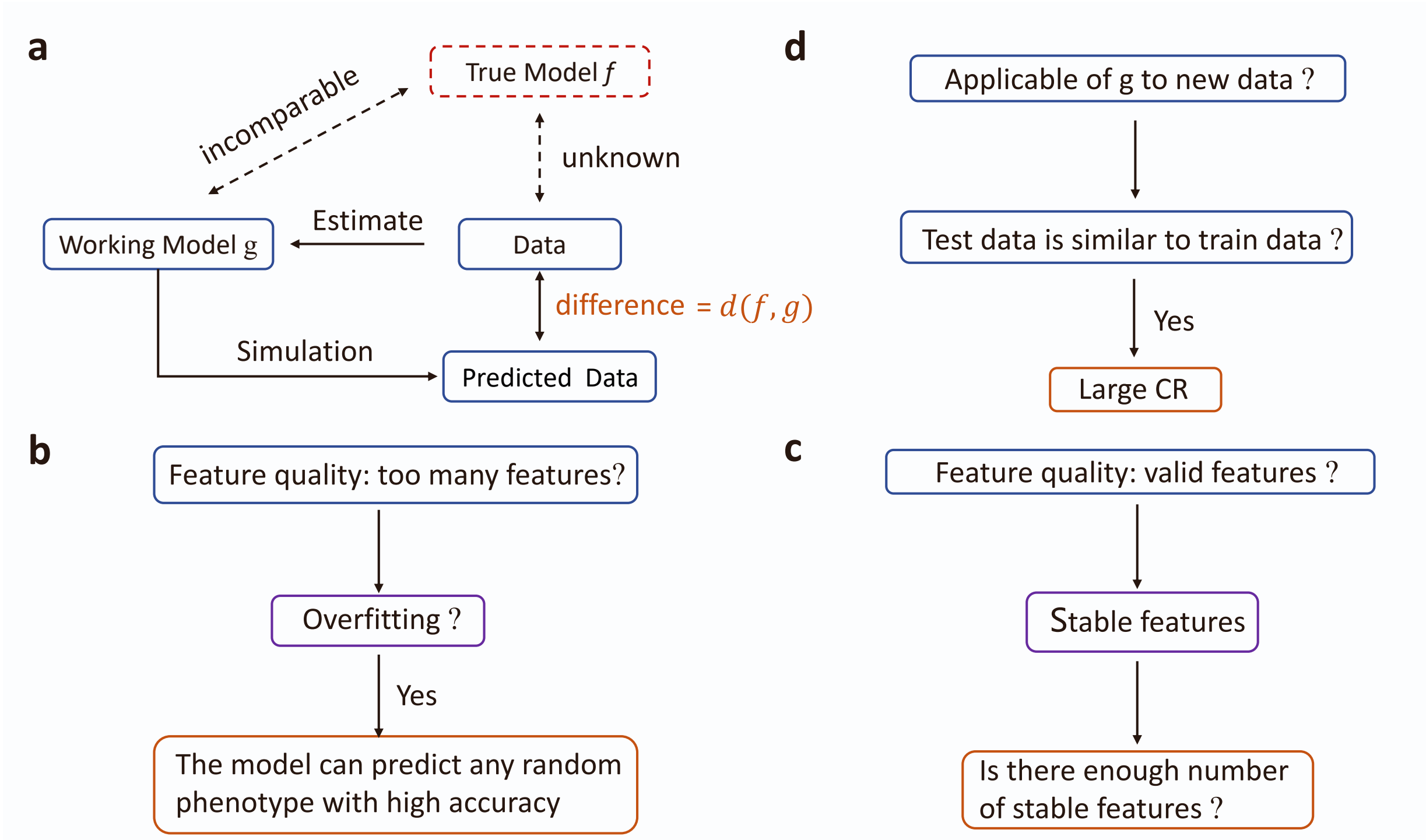

Figure S1. Conceptual summary on ideas of evaluations in mBoost. **a**, Concept of E_1 for evaluating the model structure. $d(f, g)$ is the distance between the true model f and the working model g. **b,c**, Concept of E_2(**b**), E_3(**c**) for evaluating the quality of features. **d**, Concept of E_4 on evaluating the applicability of the trained model to new dataset. Related to Fig. 1.

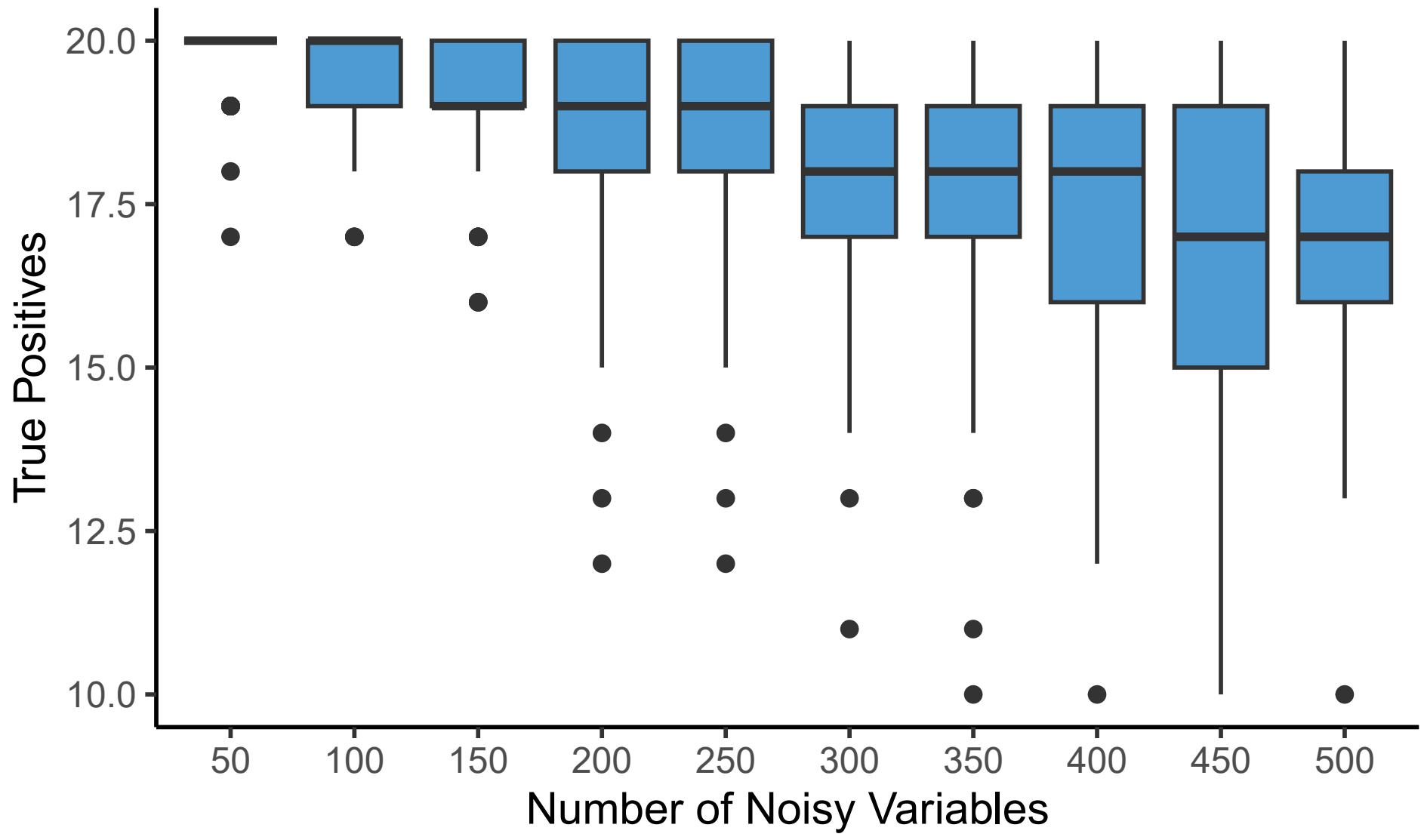

Figure S2. The feature selection capability of Lasso decreases as the number of noisy variables increases (**Methods**: Example 1). Related to Fig. 1.

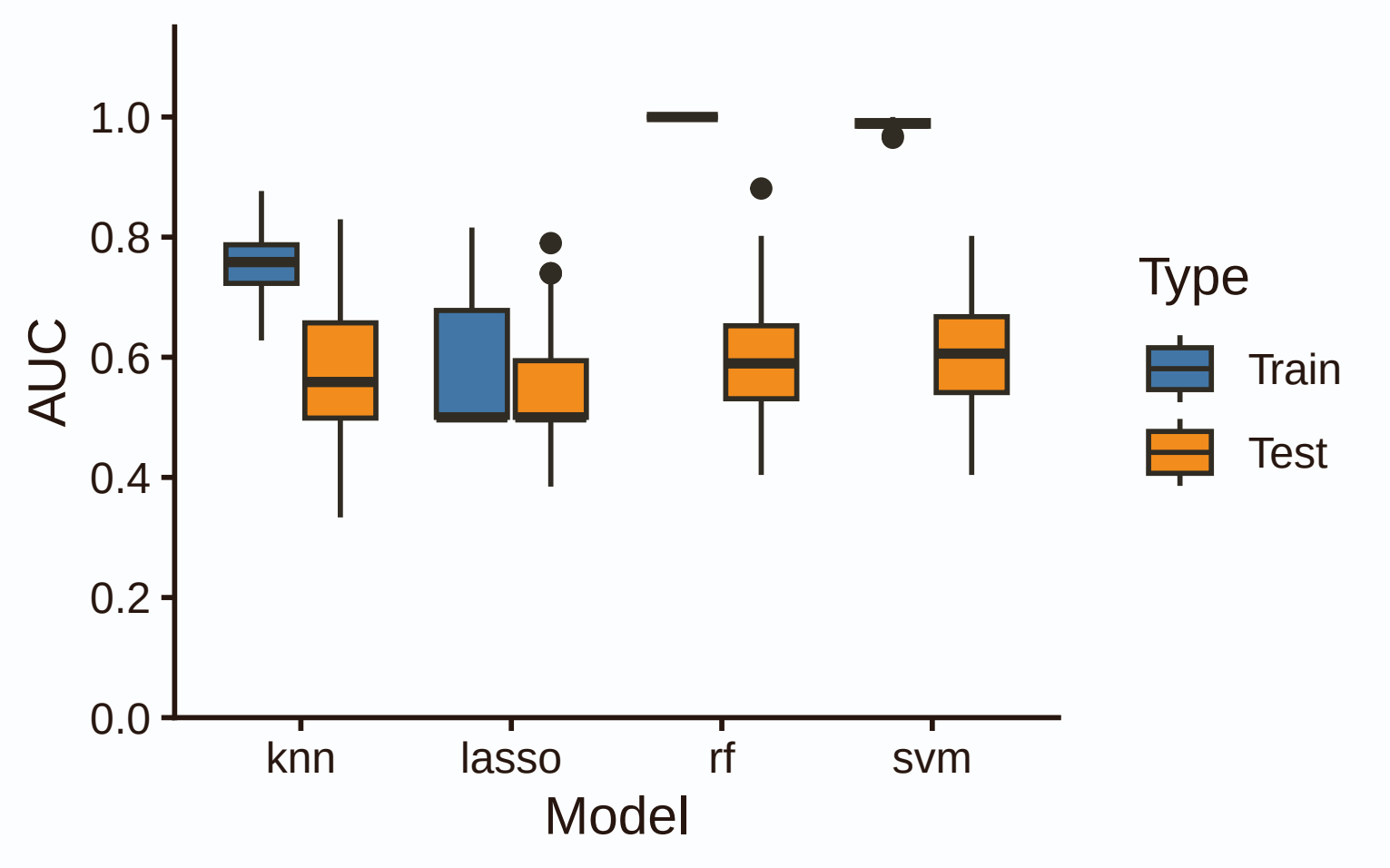

Figure S3. Modeling performance of four machine learning methods on null data. KNN and Lasso yield relatively low training AUCs, whereas Random Forest and SVM achieve near-perfect training AUCs; however, all methods produce test AUCs close to 0.5. Related to Fig. 2.

Model		KNN	Lasso	RF	SVM
Structure Improvability	I	0	0	0	0
	NI	1	1	1	1
Feature redundancy	R	0.94	0.95	1	0.95
	NR	0.06	0.05	0	0.05
Feature selection method		Wilcoxon	KS	AD	RF
Feature validity	VF	0	0	0	0
	IVF	1	1	1	1

Table S1. mBoost identifies suboptimal models and low-quality features on null data. The frequencies of the evaluation results are reported from 100 repeated simulations. mBoost consistently identified the non-improvable model structure and the low-quality features. Related to Fig. 2.

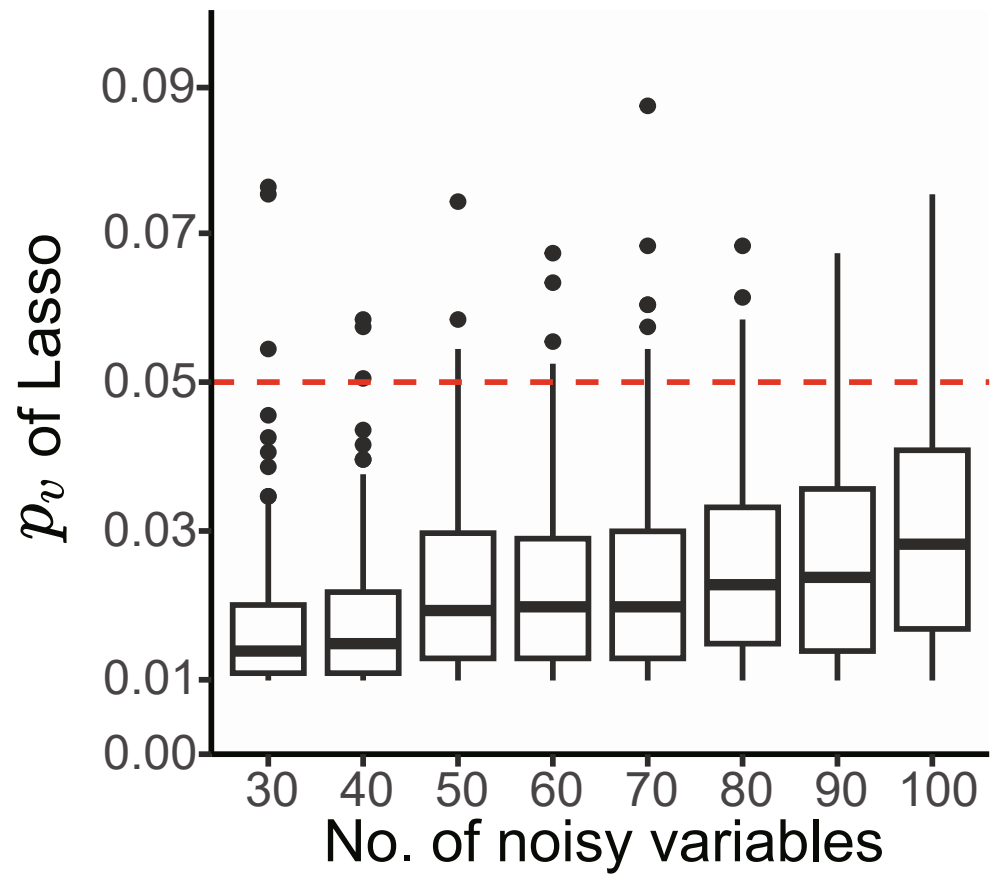

Figure S4. Lasso selects valid features in the linear logistic regression example (**Methods**: Example 4). Evaluation by mBoost revealed that in most experiments, p_v values were less than 0.05, implying that Lasso successfully identified valid features in this example. Related to Fig. 2.

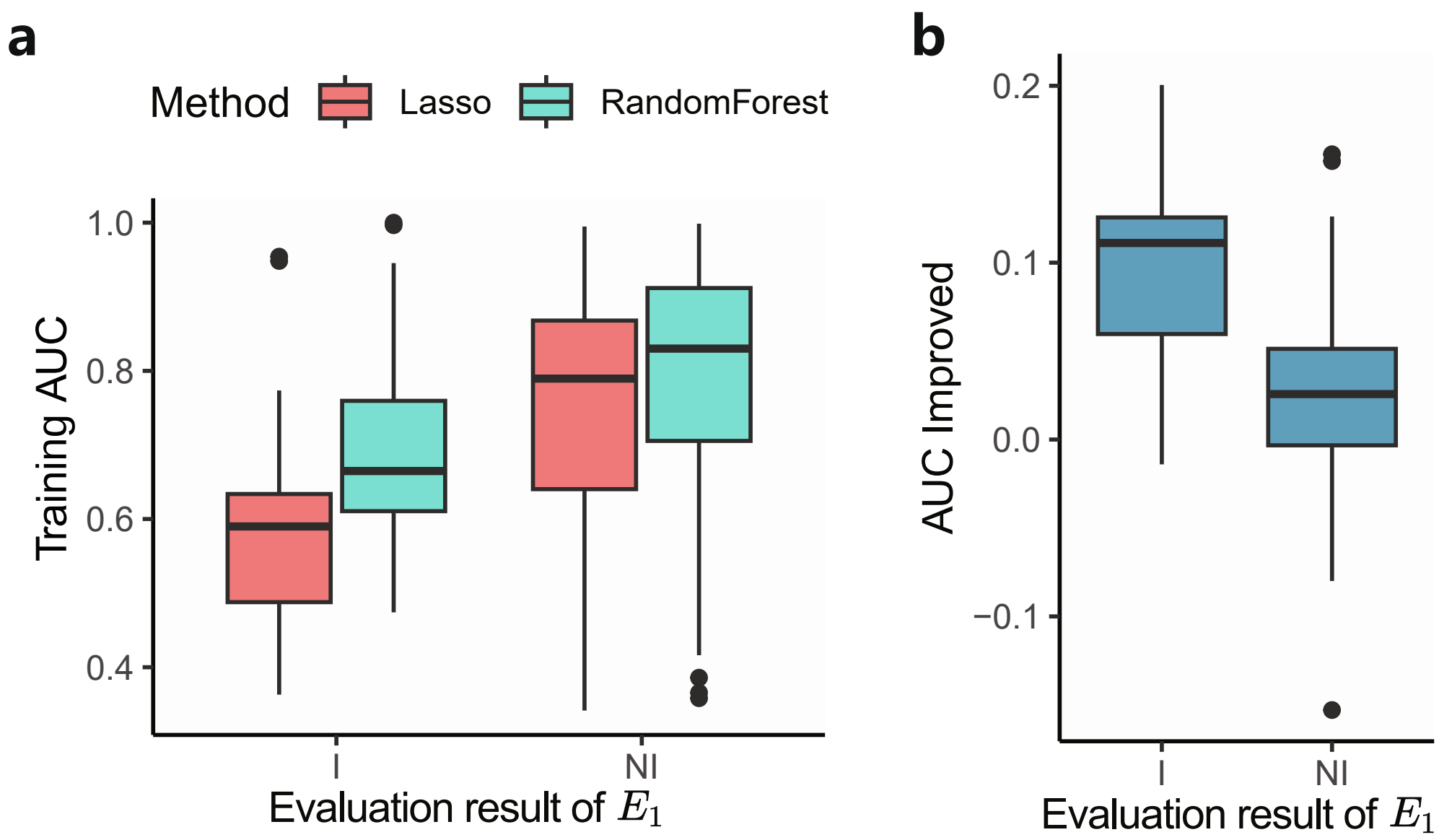

Figure S5. Internal cross-validation AUCs on the training set obtained from Lasso and RF for cohorts classified as improvable (I) and non-improvable (NI). **a**, The comparison between the training AUCs from Lasso and RF. **b**, The difference in AUCs after switching from Lasso to RF. Related to Fig. 4.

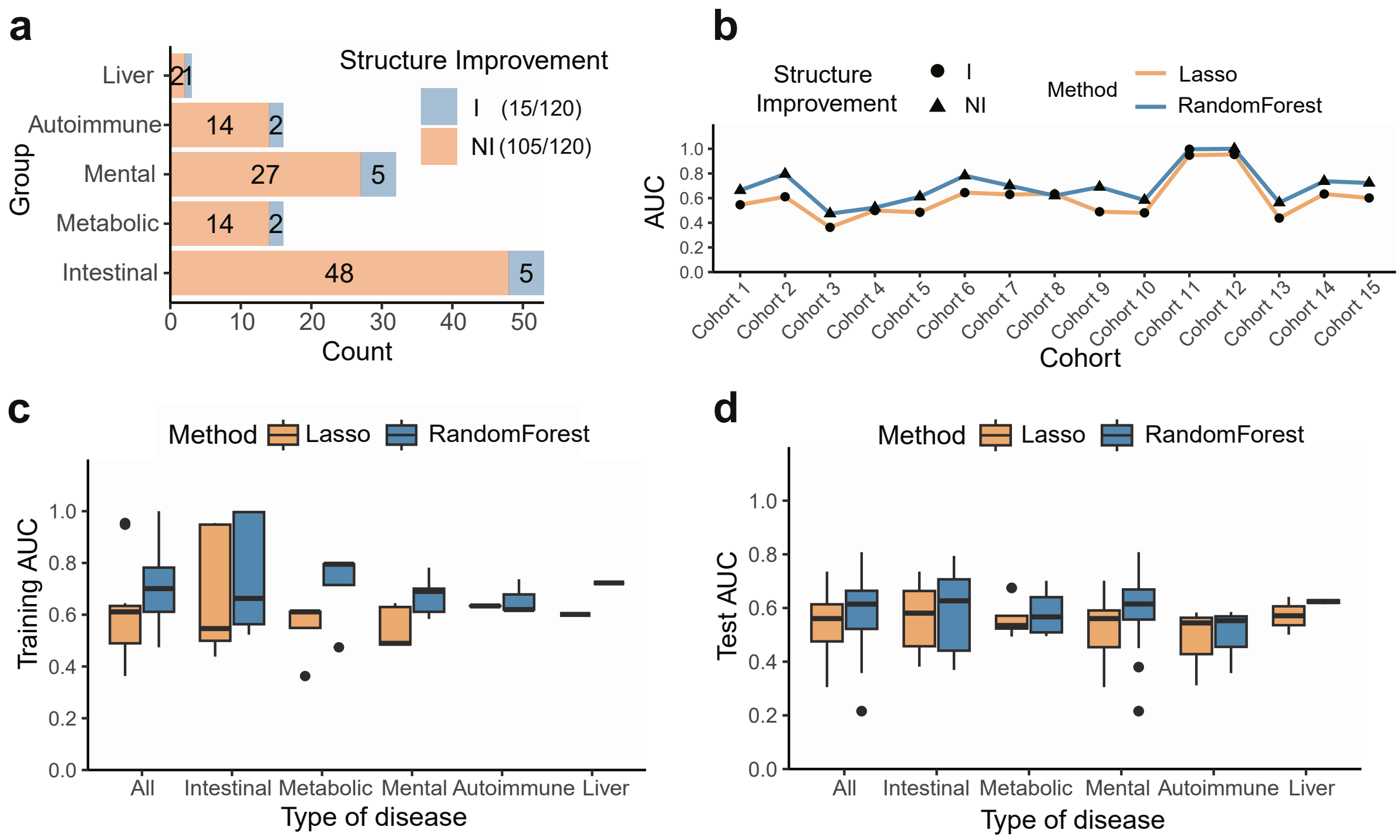

Figure S6. mBoost identifies suboptimal predictive models for different cohorts. **a**, The number of cohorts classified as I (improvable) and NI (non-improvable) under different disease categories when modeling microbiome data using Lasso. **b**, The AUC before and after switching to RF for improvable cohorts, and the corresponding determination results for E_1. **c**, Training AUCs before and after switching from Lasso to RF for the models identified as improvable in different disease category cohorts. **d**, Test AUC before and after switching from Lasso to RF for the models identified as improvable in different disease category cohorts. Related to Fig. 4.

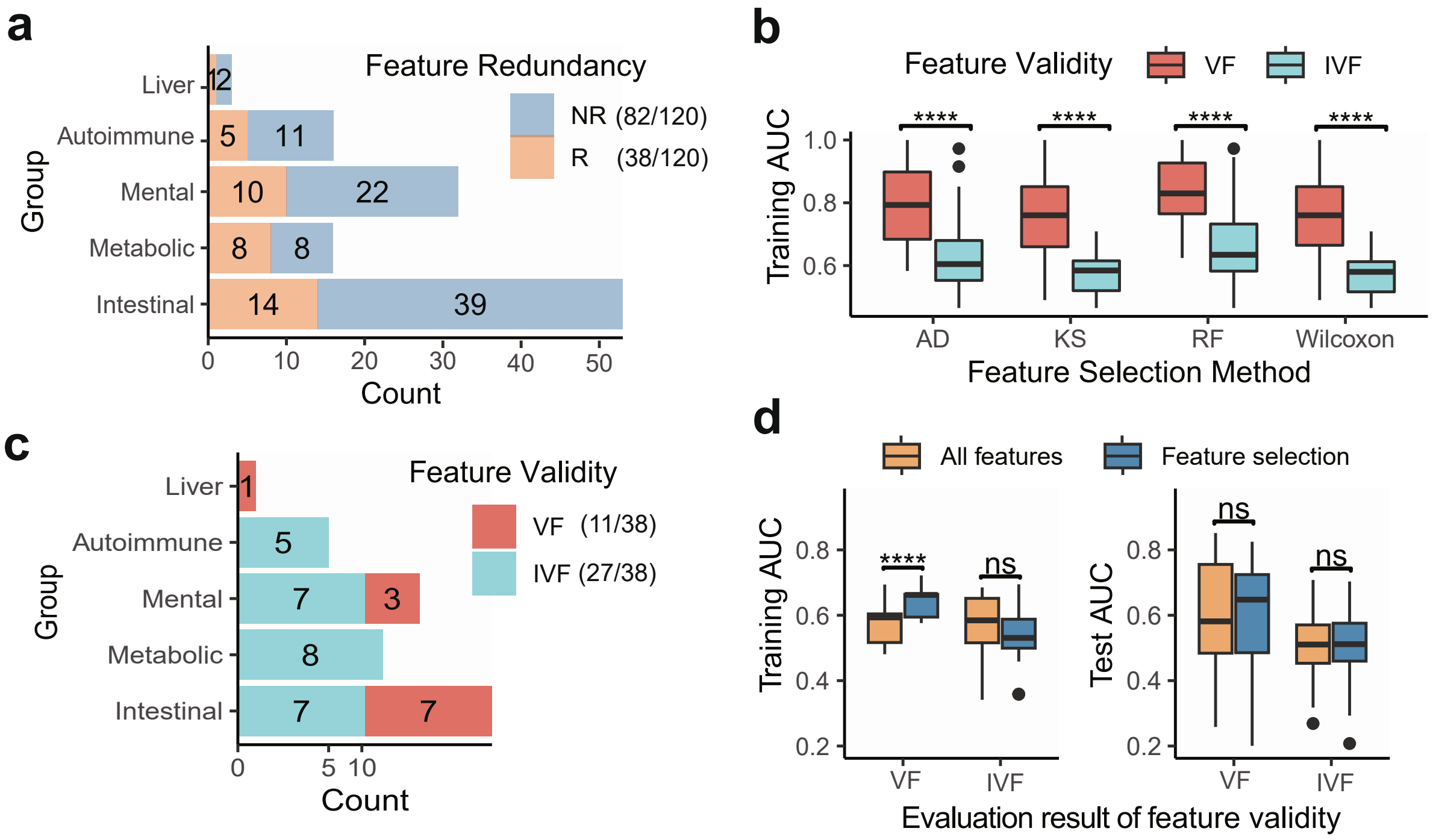

Figure S7. mBoost captures the quality of features in gut microbiome data. **a**, The number of cohorts classified as R and NR under different disease categories. **b**, Training AUCs among cohorts classified as VF and IVF. **c**, The number of cohorts classified as having valid features and those without valid features in different disease categories when using the Wilcoxon test for feature selection. **d**, Internal training AUC and external test AUC for cohorts with and without valid features. "All features" corresponds to using all of the features to build the model, and "Feature selection" corresponds to using Wilcoxon test for feature selection. For panels **b** and **d**, p values were calculated using t-tests; **** indicates $p \leq 0.0001$ and ns indicates $p > 0.05$ (sample sizes: **b**, $n = 120$; **d**, $n = 108$). Related to Fig. 4.

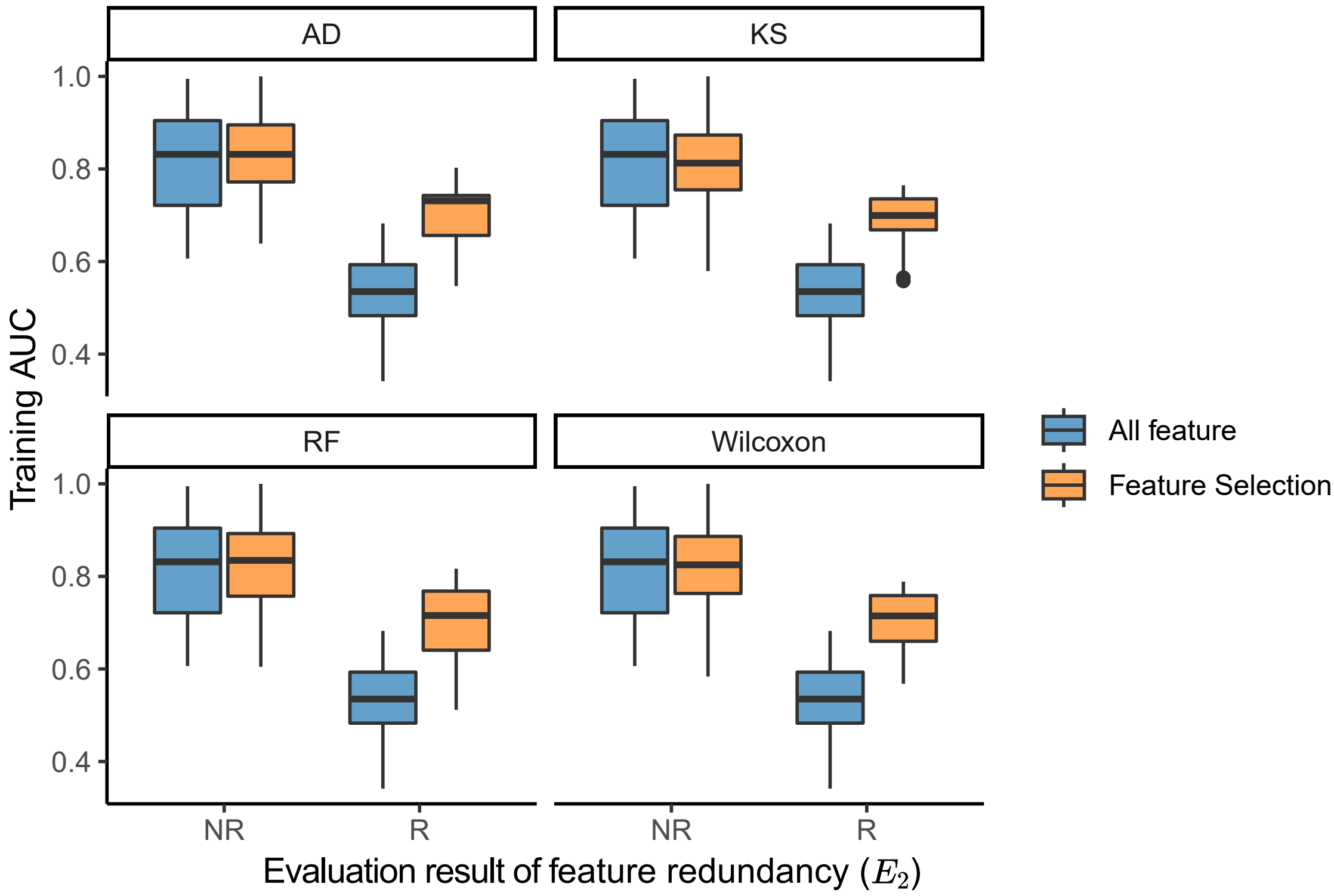

Figure S8. Training AUC before and after feature selection for cohorts classified as R and NR, where R indicates the cohorts identified as having redundant features, and NR indicates without redundant features. Four feature selection methods are used in the analysis (**Methods**). The AUC improves significantly after feature selection for R cohorts, whereas for NR cohorts the AUC shows little change before and after feature selection. "All features" means the model is trained on all features and "Feature selection" means the model is trained on selected features by different methods. Related to Fig. 4.

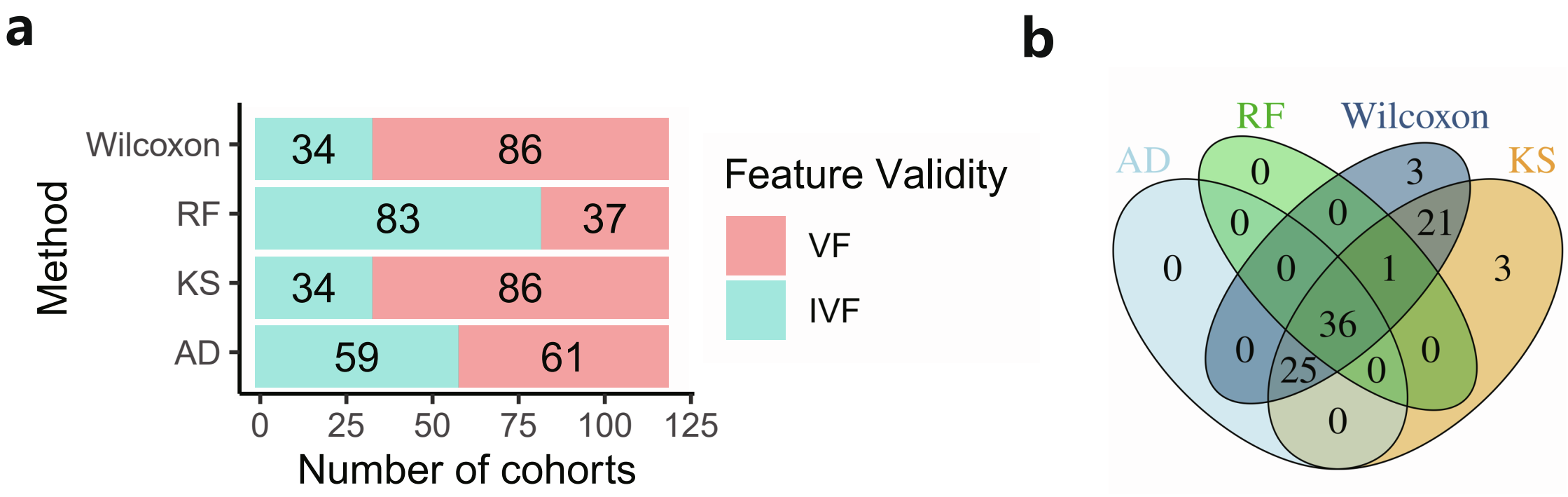

Figure S9. The distribution of cohorts identified as having valid features under four feature selection methods. **a**, The number of cohorts classified as having valid features (VF) and those without valid features (IVF). **b**, A Venn diagram illustrating the cohorts identified as having consistent features across the four feature selection methods. Related to Fig. 4.

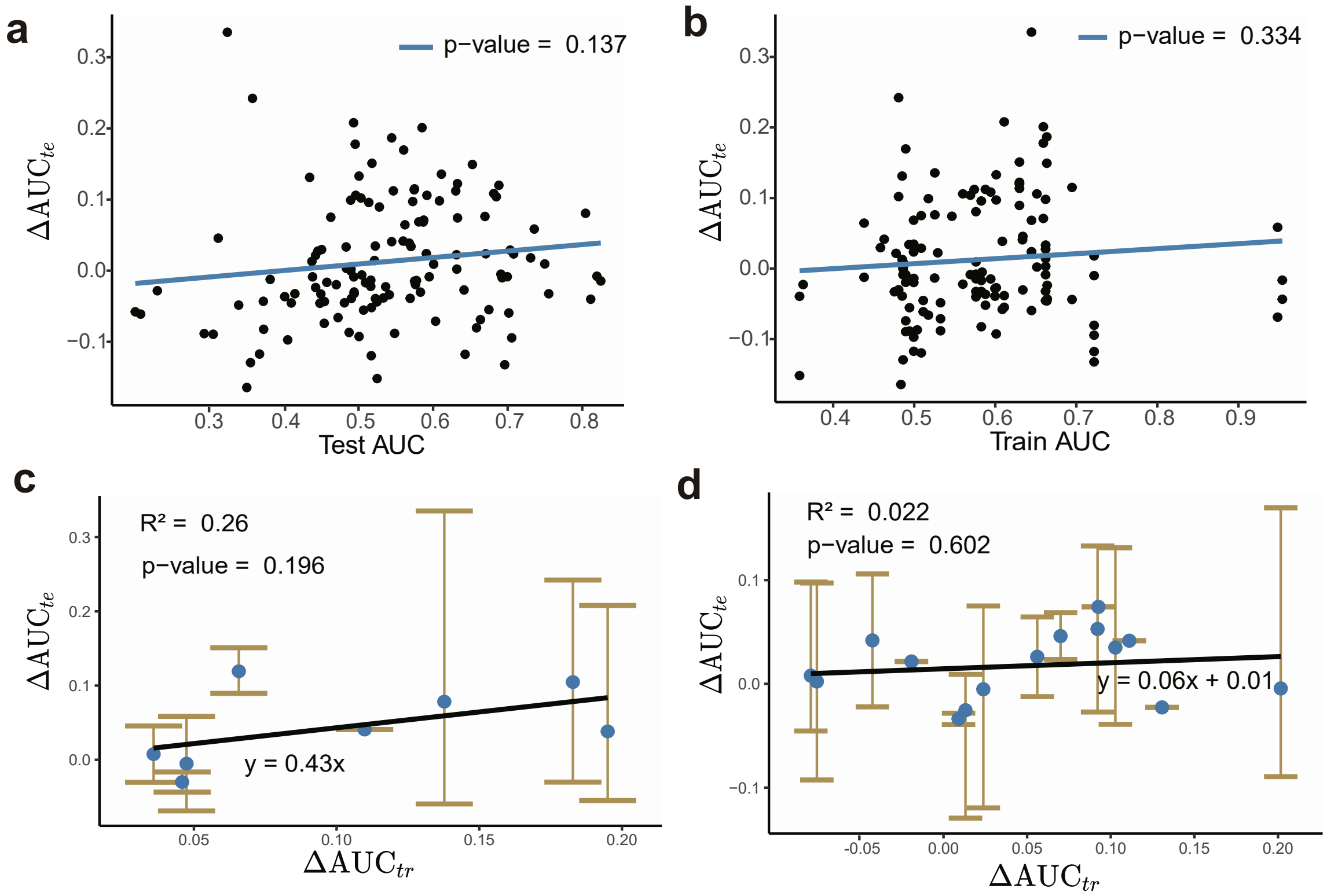

Figure S10. The relationship between ΔAUC_{te}, ΔAUC_{tr}, test AUC and training AUC, where ΔAUC_{te} is the improvement in test AUC, and ΔAUC_{tr} is the improvement in train AUC. **a,b**, The relationship between ΔAUC_{te} and test/training AUC on all cohorts identified as improvable. **c,d**, The relationship between ΔAUC_{te}, test AUC and training AUC on cohorts identified as I-NR/I-R-VF (**c**) and NI-R-IVF/I-R-IVF/NI-R-VF (**d**). P values shown in panels **a-d** were calculated as in Figure 4G using linear regression models fitted with R `lm()`; they correspond to two-sided t-tests for the slope (sample sizes: **a**, $n = 129$; **b**, $n = 129$; **c**, $n = 8$; **d**, $n = 15$). Related to Fig. 4.

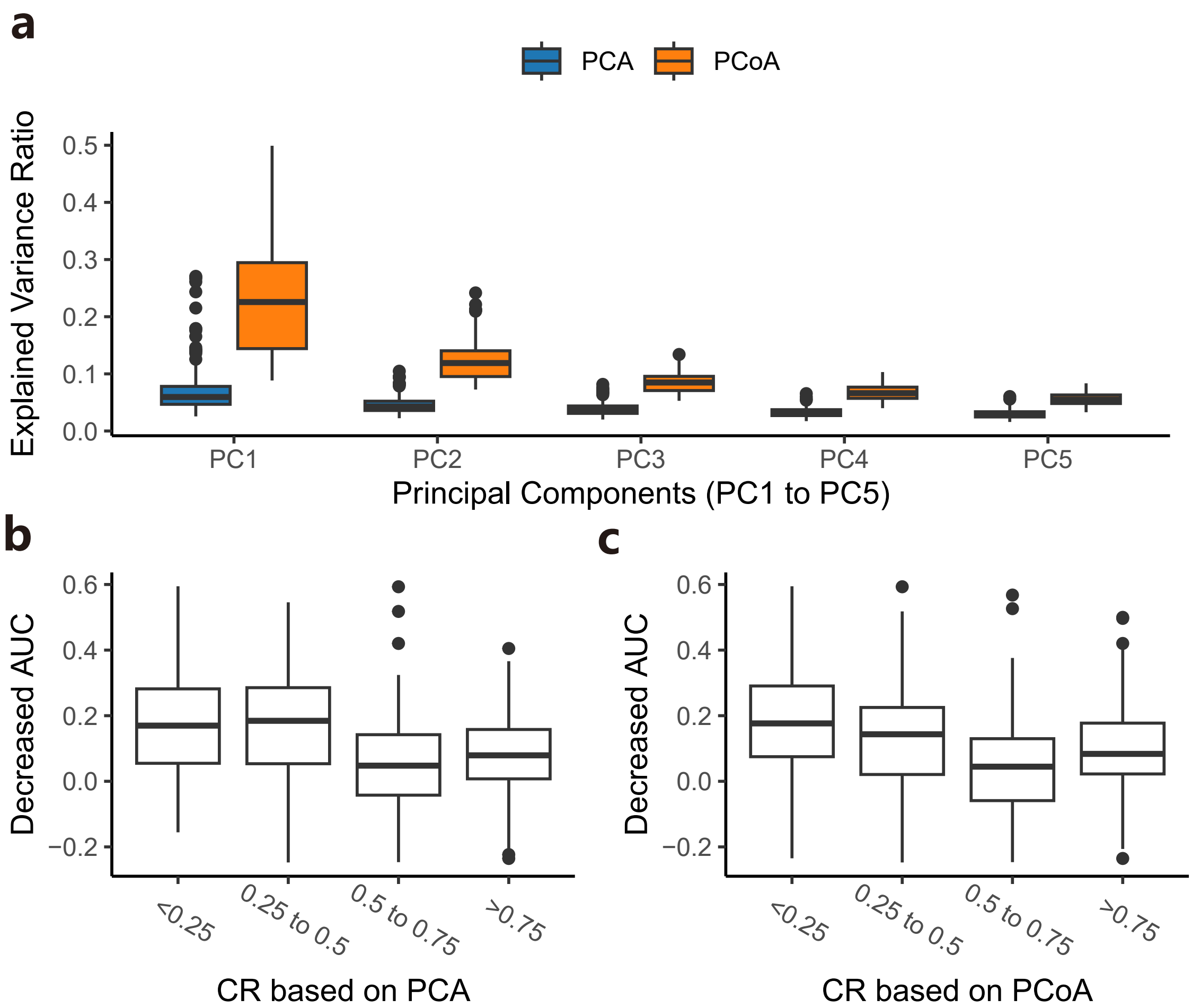

Figure S11. Coverage Ratio (CR) based on PCA and PCoA. **a**, Boxplots of the explained variance ratio for the first five principal components (PCs) from these two methods across all cohorts. **b**,**c**, The relationship between the CR based on PCA (PCoA) and the reduction in AUC. Related to Fig. 5.

Supplemental Proof

Lemma S1. *Suppose that $\boldsymbol{U}_n = (U_{n,1}, \cdots, U_{n,k})$ and $\boldsymbol{V}_n = (V_{n,1}, \cdots, V_{n,k})$ are independently sampled from multinomial distributions with parameters $(n, a_1, \cdots, a_k)$ and $(n, b_1, \cdots, b_k)$, respectively. Define the column vectors $\boldsymbol{a} = (a_1, \cdots, a_k)$ and $\boldsymbol{b} = (b_1, \cdots, b_k)$. Under the null hypothesis H_0: $\boldsymbol{a} = \boldsymbol{b}$, we have*

$$T = \sum_{i=1}^{k} \sum_{i=1}^{k} \frac{(U_{n,i} - V_{n,i})^2}{N\hat{c}_i} \rightarrow \chi^2_{k-1},$$

where $\hat{\mathbf{c}} = \frac{\boldsymbol{U}_n + \boldsymbol{V}_n}{2n}$ is the estimate of $\boldsymbol{a}$ under H_0, and $k \geq 2$.

Proof: Let $\{X_t\}_{t=1}^n$ and $\{Y_t\}_{t=1}^n$ be the observed category series of the count vectors $\mathbf{U}_n$ and $\mathbf{V}_n$, respectively. So we have $P(X_t = i) = a_i$ and $P(Y_t = i) = b_i$. Let $N = 2n$. We define

$$X_i^* = (X_{1,i}^*, \cdots, X_{N,i}^*) = (\delta_{i,X_1}, \cdots, \delta_{i,X_n}, 0, \cdots, 0),$$
$$Y_i^* = (Y_{1,i}^*, \cdots, Y_{N,i}^*) = (0, \cdots, 0, \delta_{i,Y_1}, \cdots, \delta_{i,Y_n}),$$

where $\delta_{i,j} = I(i = j)$.

Let $Z_i = U_{n,i} - V_{n,i} = \sum_{k=1}^{N} X_{k,i}^* - \sum_{k=1}^{N} Y_{k,i}^*$, under H_0, we have $E(Z_i) = 0, \text{Var}(Z_i) = 2na_i(1 - a_i), \text{Cov}(Z_i, Z_j) = -2na_ia_j$. So, by the multivariate central limit theorem, we have

$$\frac{1}{\sqrt{2n}}\mathbf{Z} = \frac{\mathbf{U}_n - \mathbf{V}_n}{\sqrt{2n}} \xrightarrow{d} N(0, \Sigma),$$

where $\sigma_{i,j}$, the (i,j)-th element of Σ, is equal to $a_i(\delta_{ij} - a_j)$. Since $\hat{\mathbf{c}} \xrightarrow{p} \mathbf{a}$, we have

$$\frac{\mathbf{U}_n - \mathbf{V}_n}{\sqrt{2n\hat{\mathbf{c}}}} \xrightarrow{d} N(0, I_k - \sqrt{\mathbf{a}}\sqrt{\mathbf{a}}').$$

Thus, we have $T \rightarrow \chi^2_{k-1}$, as $n \rightarrow +\infty$.

Proof of Theorem 1

Denote $\hat{p}_{ij} = P_{ij}/n$, $\hat{q}_{ij} = Q_{ij}/n$, and $\hat{r}_{ij} = \hat{p}_{ij} + \hat{q}_{ij}$ as the estimates of p_{ij}, q_{ij} and r_{ij}, respectively. Let $\Delta_{ij} = p_{ij} - q_{ij}$, and let $\hat{\Delta}_{ij} = \hat{p}_{ij} - \hat{q}_{ij}$ be its estimate.

It is well known that for $\hat{p}_{ij}$, $\hat{q}_{ij}$, we have

$$P(|\hat{p}_{ij} - p_{ij}| > \epsilon) \leq 2e^{-n\epsilon^2/2}, \ \ P(|\hat{q}_{ij} - q_{ij}| > \epsilon) \leq 2e^{-n\epsilon^2/2}.$$

Thus, we have,

$$P(|\hat{\Delta}_{ij} - \Delta_{ij}| > \epsilon) \leq 4e^{-n\epsilon^2/8}, \ \ P(|\hat{r}_{ij} - r_{ij}| > \epsilon) \leq 4e^{-n\epsilon^2/8}.$$

For convenience, let S be the local statistic defined in equation (3), and let S^p be its population version defined in equation (4).

$$\begin{aligned}
|S - S^p| &= n|\sum_{i=1}^{2}\sum_{j=1}^{2}\frac{(\hat{p}_{ij} - \hat{q}_{ij})^2}{\hat{r}_{ij}} - \sum_{i=1}^{2}\sum_{j=1}^{2}\frac{(p_{ij} - q_{ij})^2}{r_{ij}}| \\
&\leq n\sum_{i=1}^{2}\sum_{j=1}^{2}|\frac{(\hat{p}_{ij} - \hat{q}_{ij})^2}{\hat{r}_{ij}} - \frac{(p_{ij} - q_{ij})^2}{r_{ij}}| \\
&= n\sum_{i=1}^{2}\sum_{j=1}^{2}|\frac{(\hat{p}_{ij} - \hat{q}_{ij})^2}{\hat{r}_{ij}} - \frac{(\hat{p}_{ij} - \hat{q}_{ij})^2}{r_{ij}} + \frac{(\hat{p}_{ij} - \hat{q}_{ij})^2}{r_{ij}} - \frac{(p_{ij} - q_{ij})^2}{r_{ij}}| \\
&\leq n\sum_{i=1}^{2}\sum_{j=1}^{2}\frac{\hat{\Delta}_{ij}^2}{\hat{r}_{ij}r_{ij}}|\hat{r}_{ij} - r_{ij}| + \frac{|\hat{\Delta}_{ij}^2 - \Delta_{ij}^2|}{r_{ij}} \\
&\leq n\sum_{i=1}^{2}\sum_{j=1}^{2}\frac{|1 - r_{ij}/\hat{r}_{ij}|}{\eta} + \frac{2|\hat{\Delta}_{ij} - \Delta_{ij}|}{\eta}
\end{aligned}$$

Define the events $E_1 = \{|\hat{\Delta}_{ij} - \Delta_{ij}| > \epsilon\}$ and $E_2 = \{|\hat{r}_{ij} - r_{ij}| > \epsilon\}$. Now, on $E_1^c \bigcap E_2^c$, we have

$$|S - S^p| \leq \frac{4n\epsilon}{\eta}(\frac{1}{\eta} + 2). \tag{S1}$$

Finally, let $\delta = \frac{4n\epsilon}{\eta}(\frac{1}{\eta} + 2)$, we have

$$P(|S - S^p| > \delta) \leq 1 - P(E_1^c \bigcap E_2^c) \leq P(E_1) + P(E_2) = 8e^{-n\epsilon^2/8}. \tag{S2}$$

Thus, $P(|\text{MAC}(D, D^*) - \text{MAC}^p(D, D^*)| > \delta) \leq 16n^2e^{-n\epsilon^2/8}$, and the other two conclusions follow directly.
